# A Multidimensional Engineering Strategy Reprograms Microglia via Targeted and Sustained‐Release Extracellular Vesicles for Spinal Cord Injury Repair

**DOI:** 10.1002/advs.77117

**Published:** 2026-08-13

**Authors:** Wu Xiong, Minhao Liu, Mingming Zheng, Jizhou Jia, Ziyan Zhu, Guang Kong, Jie Liu, Juan Wang, Wenbo Li, Qingyuan Wang, Qian Zhu, Peiran Chan, Tao Qin, Jiayang Wu, Yongjie Zhang, Chunming Tang, Cong Li, Jin Fan

**Affiliations:** ^1^ Department of Orthopedics The First Affiliated Hospital of Nanjing Medical University Nanjing Jiangsu China; ^2^ Department of human anatomy School of Basic Medicine Nanjing Medical University Nanjing Jiangsu China; ^3^ Department of Orthopaedics Fourth Military Medical University Xi'an Shaanxi China; ^4^ Department of Orthopedics The Affiliated Taizhou People's Hospital of Nanjing Medical University Taizhou School of Clinical Medicine Nanjing Medical University Taizhou Jiangsu China; ^5^ Department of Pharmaceutics School of Pharmacy Nanjing Medical University Nanjing Jiangsu China

**Keywords:** mesenchymal stem cell, microglia, neuroinflammation, remyelination, spinal cord injury

## Abstract

Spinal cord injury (SCI) induces neuroinflammation predominantly mediated by microglia, thereby establishing a detrimental milieu that impedes neurological recovery. Extracellular vesicles (EVs) derived from umbilical cord mesenchymal stem cells (UCMSCs) possess considerable therapeutic potential; however, their clinical translation is constrained by insufficient bioactivity, poor targeting specificity, and uncontrolled release kinetics. Here, we present a multidimensional engineering strategy that overcomes these barriers synergistically. Tetramethylpyrazine (TMP)‐pretreated extracellular vesicles (TEVs) are enriched with anti‐inflammatory and pro‐regenerative factors in their cargo, while Angiopep‐2 (Ang2) peptide‐modified TEVs (Ang‐TEVs) confer significantly enhanced microglial targeting. A reactive oxygen species (ROS)‐responsive hyaluronic acid (HA)‐phenylboronic acid (PBA)/polyvinyl alcohol (PVA) hydrogel serves as an intelligent depot for sustained, on‐demand Ang‐TEVs release at the lesion site. This construct, Ang‐TEVs@Gel, demonstrated robust lesion accumulation and selective microglial uptake. It delivered miR‐664a‐3p, which suppressed PIK3CA to attenuate PI3K‐AKT‐mTOR signaling and unleash autophagic flux, reprogramming microglia toward a reparative state that enhanced myelin debris clearance and quelled inflammation. Consequently, axonal regeneration and remyelination were markedly improved, driving significant motor recovery in SCI mice. By integrating preconditioning, active targeting, and stimuli‐responsive biomaterials, this strategy provides an elegant blueprint for engineering EV‐based therapies to repair the injured central nervous system.

## Graphical abstract

A multifunctional EV‐based nanoplatform (Ang‐TEVs@Gel) was engineered via preconditioning, surface targeting, and ROS‐responsive hydrogel encapsulation to reprogram microglia. This system restored autophagy via miR‐664a‐3p/PIK3CA axis, cleared myelin debris, resolved neuroinflammation, and promoted axon remyelination, ultimately achieving robust motor recovery in spinal cord injury mice.

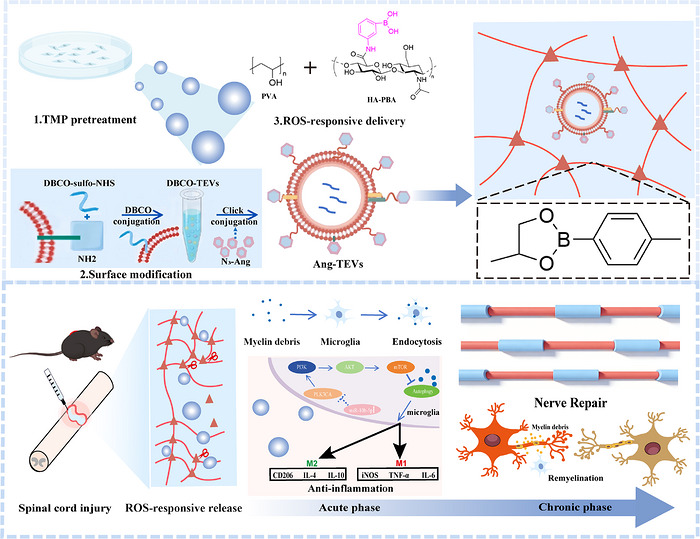

**Abbreviations**
3‐MA: 3‐methyladenine
Ang2: Angiopep‐2
Ang‐TEVs: Angiopep‐2 peptide‐modified TEVs
BMS: Basso Mouse Scale
DAMPs: Damage‐Associated Molecular Patterns
DLS: Dynamic light scattering
EVs: Extracellular vesicles
GO: Gene Ontology
H&E: Hematoxylin and Eosin
HA: Hyaluronic acid
LFB: Luxol Fast Blue
LRP1: Low‐density lipoprotein receptor‐related protein 1
MEP: Motor evoked potential
miRNAs: microRNAs
mTORC1: Mammalian target of rapamycin complex 1
MUT: Mutant
OPCs: Oligodendrocyte precursor cells
PBA: Phenylboronic acid
PVA: Polyvinyl alcohol
RISC: RNA‐induced silencing complex
ROS: Reactive oxygen species
SCI: Spinal cord injury
SEM: Scanning electron microscopy
SnRNA‐seq: Single‐nucleus RNA sequencing
TEM: Transmission electron microscopy
TEVs: Tetramethylpyrazine‐pretreated extracellular vesicles
TMP: Tetramethylpyrazine
UCMSCs: Umbilical cord mesenchymal stem cells
WB: Western blot
WT: Wild‐type

## 1 Introduction

The pathological process following spinal cord injury (SCI) is complex, involving two stages known as primary injury and secondary injury. The primary injury denotes the initial mechanical trauma, which instigates the rapid release of Damage‐Associated Molecular Patterns (DAMPs). These DAMPs promptly activate resident microglia and facilitate the infiltration of peripheral immune cells into the lesion site [1]. The secondary injury comprises a series of molecular and cellular events that ensue following the primary damage and represents a critical temporal window that dictates the neurological functional prognosis after SCI [2]. Neuroinflammation serves as a central driving mechanism in the initiation and progression of this secondary phase [3]. Within this context, the dynamic phenotypic transition of microglia is of particular importance [4, 5]. Initially, microglia may exert protective functions through the clearance of myelin debris. However, as the pathology advances, sustained polarization toward a pro‐inflammatory phenotype results in the excessive release of inflammatory mediators, including TNF‐α and IL‐1β. This leads to a pronounced “inflammatory storm”, which further compromises the blood‐spinal cord barrier, intensifies oxidative stress, promotes apoptosis of neurons and glial cells, and ultimately contributes to the formation of an inhibitory glial scar–‐severely impeding neural regeneration and functional recovery [6, 7, 8]. Consequently, modulating the polarization balance of microglia, toward an anti‐inflammatory and pro‐reparative phenotype has emerged as a pivotal scientific challenge in the treatment of SCI.

In recent years, the synergistic integration of engineering modification strategies and intelligent delivery systems has emerged as a promising breakthrough avenue [9, 10]. Engineered extracellular vesicles (EVs) refer to EVs or their parental cells that are modified via physical, chemical, or biological approaches to enhance their intrinsic functions or endow them with novel therapeutic attributes [11, 12]. Parental cell engineering specifically entails regulating the biological state of EV‐producing cells through gene editing or conditional pretreatment, thereby altering the composition of EV cargo [13]. Tetramethylpyrazine (TMP), the primary bioactive alkaloid derived from Ligusticum chuanxiong, manifests a series of physiological functions, including antioxidative, anti‐inflammatory, anti‐apoptotic, and neuroprotective effects [14, 15]. TMP has demonstrated therapeutic efficacy in various neurological disorders, such as ischemic stroke, spinal cord injury, and neuropathic pain [16, 17, 18]. Targeted modification represents another crucial aspect of EV engineering, which involves conjugating tissue‐specific peptides or ligands onto the EV surface using genetic engineering, chemical conjugation, or membrane fusion techniques. This approach facilitates lesion‐specific accumulation of EVs while minimizing off‐target effects [18, 19]. Angiopep‐2 (Ang2), a bioactive peptide with high affinity for low‐density lipoprotein receptor‐related protein 1 (LRP1), possesses potent cell‐penetrating capabilities [20]. Following spinal cord injury, the expression of LRP1 in microglia is significantly upregulated, enabling Ang2‐modified EVs to achieve enhanced accumulation in microglia at the injury site via ligand‐receptor‐mediated active targeting mechanisms [21].

Regarding delivery system design, hydrogels‐three‐dimensional networks formed by physical or chemical cross‐linking of hydrophilic polymers‐are capable of absorbing and retaining large volumes of water. Their unique “soft and moist” mechanical properties closely mimic those of native biological tissues such as the spinal cord and brain, and their physicochemical characteristics (e.g., pore size, degradation rate, mechanical strength) are readily adjustable, rendering them indispensable materials in biomedical applications [22, 23, 24]. Reactive oxygen species (ROS)‐responsive hydrogels, in particular, are designed to dynamically respond to the elevated ROS microenvironment of injured tissues. Through reversible cleavage of phenylboronate ester linkages, these hydrogels enable on‐demand drug release, thereby preserving therapeutic bioactivity and avoiding premature degradation [25].

In this study, we propose a triple‐component engineering strategy to construct a composite therapeutic system, Ang‐TEVs@Gel, by integrating three key components: enhancing EV bioactivity through TMP pretreatment, promoting microglia‐specific targeted accumulation via Ang2 surface modification, and enabling spatiotemporally controlled release with a ROS‐responsive hydrogel matrix.

## 2 Results

### 2.1 Extraction and Characterization of TEVs

**FIGURE 1:**
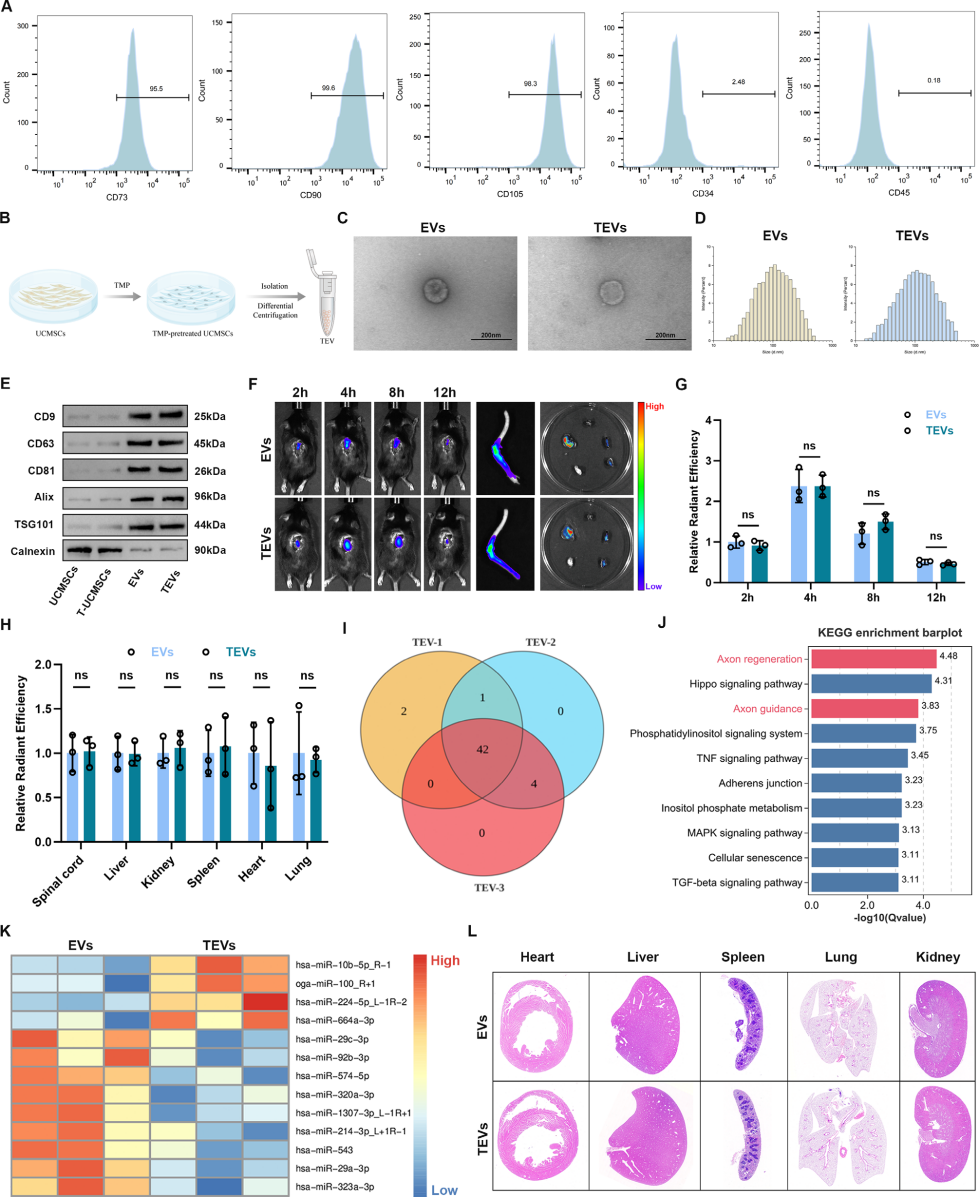
Characterization of EVs and TEVs. (A) Flow cytometry analysis of mesenchymal stem cell markers. (B) Schematic diagram illustrating the procedure for the isolation of extracellular vesicles from UCMSCs following TMP pretreatment. (C) Representative TEM images showing the characteristic morphology of both EVs and TEVs. (D) Size distribution profiles of EVs and TEVs as determined by DLS. (E) WB analysis of extracellular vesicles marker proteins (Alix, CD9, CD63, CD81, TSG101) and the negative marker Calnexin in both EVs and TEVs. (F–H) Representative in vivo and ex vivo organ imaging, along with fluorescence intensity quantification, of mice treated with EVs or TEVs (n = 3). (I) Venn diagram identifying 42 commonly highly expressed miRNAs across all TEVs samples, defined as miRNAs with read counts >1000 (n = 3). (J) KEGG pathway enrichment analysis of the predicted target genes of the 42 core highly expressed miRNAs. (K) Differential analysis of miRNAs in TEVs and EVs (n = 3). (L) H&E staining of heart, liver, spleen, lungs and kidneys for safety evaluation.

Flow cytometry identification outcomes demonstrated that umbilical cord mesenchymal stem cells (UCMSCs) manifested positive expression of markers CD90, CD105, and CD73, while presenting negative expression for CD45 and CD34 (Figure 1A). Subsequent to the pretreatment of umbilical cord mesenchymal stem cells with tetramethylpyrazine, extracellular vesicles were extracted (Figure 1B). Transmission electron microscopy (TEM) observations disclosed that both tetramethylpyrazine‐pretreated extracellular vesicles (TEVs) and conventional EVs displayed analogous cup‐shaped configurations (Figure 1C). Dynamic light scattering (DLS) further verified that TEVs and EVs nanoparticles possessed similar size distribution traits (Figure 1D). Western blot (WB) analysis ascertained the existence of key extracellular vesicle markers Alix, CD9, CD63, CD81, and TSG101 in both EVs and TEVs, while the absence of the non‐extracellular vesicle biomarker Calnexin corroborated the purity of the samples (Figure 1E). In vivo and ex vivo organ imaging results indicated that the pretreatment did not modify the in vivo distribution of the vesicles (Figure 1F–H). Given that microRNAs (miRNAs) are the principal functional messenger molecules of extracellular vesicles, this study carried out high‐throughput miRNA sequencing analysis. All miRNAs were ranked according to sequencing read counts, with miRNAs having read counts surpassing 1000 in each sample defined as highly expressed miRNAs. Through taking the intersection of highly expressed miRNAs across all TEVs samples, 42 commonly highly expressed miRNAs were ultimately identified (Figure 1I and Table S1). KEGG pathway analysis of the target genes of these highly expressed miRNAs revealed that these 42 core miRNAs predominantly regulate signaling pathways associated with axon regeneration and axon guidance (Figure 1J), indicating that TEVs possess excellent potential for promoting neural regeneration. Moreover, differential analysis demonstrated that, in comparison to EVs, the expression of multiple miRNAs in TEVs was significantly modulated. This result suggests that the miRNA changes induced by TMP pretreatment may be the intrinsic determinants responsible for the enhanced functional performance of TEVs (Figure 1K). To investigate whether TMP pretreatment affects the biocompatibility of EVs, major organs were harvested and subjected to Hematoxylin and Eosin (H&E) staining analysis. The results demonstrated no significant pathological alterations in the major organs of either treatment group (Figure 1L).

### 2.2 Preparation of Ang‐TEVs via the Click Chemistry Method

**FIGURE 2:**
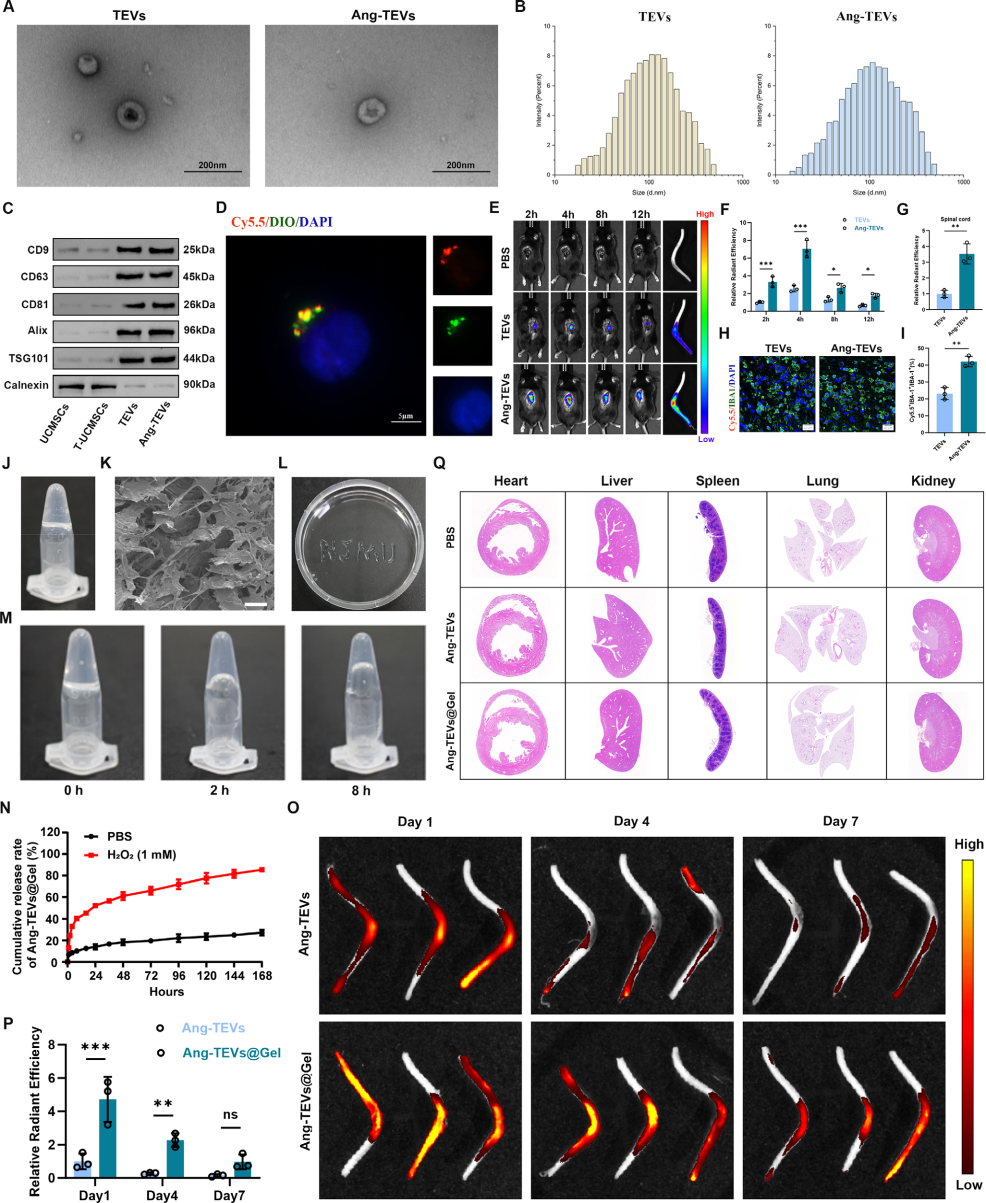
Preparation, characterization, and ROS‐responsive hydrogel encapsulation of Ang‐TEVs. (A) Representative TEM images showing the characteristic morphology of both TEVs and Ang‐TEVs. Scale bar, 200 nm. (B) Size distribution profiles of TEVs and Ang‐TEVs as determined by DLS. (C)Western blot analysis confirming the expression of key markers (CD9, CD63, CD81, Alix, TSG101) in both TEVs and Ang‐TEVs. (D) The peptide was tagged at its C‐terminus with the Cy5.5 fluorophore, while the modified EVs were counterstained with DiO. Subsequent confocal microscopy was employed to assess the colocalization of the two fluorescent signals. (E) Representative in vivo and ex vivo spinal cord fluorescence images at various time points post‐intravenous injection of Cy5.5‐labeled TEVs or Ang‐TEVs in SCI mouse models. (F,G) Quantitative analysis of fluorescence intensity of in vivo and ex vivo spinal cord (n = 3). (H,I) Colocalization analysis of Cy5.5‐labeled TEVs and Ang‐TEVs with microglia in the core region of spinal cord injuries (n = 3). Scale bar, 20 µm. (J) Macroscopic images demonstrating the gel's mechanical stability and self‐supporting capacity through an inversion test. (K) Representative SEM image of the lyophilized Ang‐TEVs@Gel. Scale bar, 100 µm. (L) Injectable property of Ang‐TEVs@Gel. (M) Fluidity test illustrating the ROS‐responsive degradation of the hydrogel. (N) In vitro cumulative release profile of EVs from Ang‐TEVs@Gel over 7 days under PBS or ROS conditions (n = 3). (O) Representative ex vivo fluorescence images of spinal cord tissues at days 1, 4, and 7 post‐implantation of Dil‐labeled Ang‐TEVs or Ang‐TEVs@Gel. (P) Quantitative analysis of the fluorescence intensity at the injury site over time (n = 3). (Q) H&E staining of heart, liver, spleen, lungs, and kidneys for safety evaluation.

Our previous studies have confirmed that the Ang2 peptide primarily targets microglia after spinal cord injury [21, 26]. In order to enhance the targeting ability and penetrability of TEVs toward the microglia in spinal cord injury area, this research conjugated the Ang2 peptide to the surface of TEVs, thereby preparing Ang‐TEVs. Analyses using TEM and DLS indicated that peptide modification did not alter the morphology or size distribution of the extracellular vesicles (Figure 2A,B). Western blot analysis verified that both groups of extracellular vesicles expressed key positive markers: CD9, CD63, CD81, Alix, and TSG101 (Figure 2C). To validate the peptide modification, we conjugated Cy5.5 to the C‐terminus of the Ang2 peptide and stained the engineered extracellular vesicles with DiO. Confocal laser scanning microscopy revealed marked colocalization of the Cy5.5 and DiO signals, suggesting the association of the Ang2 peptide with the EV membranes (Figure 2D). To evaluate the specificity of Ang‐TEVs in targeting microglia in the injured spinal cord, Cy5.5‐labeled extracellular vesicles from both groups were administered intravenously via the tail vein in a mouse model of spinal cord injury. In vivo imaging was utilized to dynamically monitor changes in fluorescence intensity within the spinal cord over time. The imaging results revealed a pronounced accumulation of fluorescent signals at the injury site, which increased progressively, peaked at 4 h post‐injection, and subsequently declined. Notably, the Ang‐TEVs group displayed consistently higher fluorescence accumulation across all observed time points compared to the control group, suggesting that Ang2 peptide modification significantly enhances the targeting efficiency and tissue penetration capacity of TEVs toward the spinal cord lesion. Further fluorescence signal analysis and quantification of isolated spinal cord tissues confirmed that Ang‐TEVs exhibited a significantly greater targeting affinity for the injury site relative to unmodified TEVs (Figure 2E–G), but did not significantly alter visceral organ distribution patterns (Figure S1A,B). Subsequent immunofluorescence staining also demonstrated stronger Cy5.5 fluorescence signals in the Ang‐TEVs‐treated group, corroborating the in vivo imaging findings. Additionally, Ang2 peptide modification significantly enhanced the active targeting ability of TEVs to microglia (Figure 2H,I).

### 2.3 Construction of Ang‐TEVs@Gel and its Release Pattern

To accomplish the responsive delivery of Ang‐TEVs, this study successfully fabricated a ROS‐responsive hydrogel via cross‐linking through dynamic phenylboronic ester bonds and encapsulated Ang‐TEVs within it. The mixed solution of 3‐aminophenylboronic acid (PBA)‐modified hyaluronic acid (HA) and polyvinyl alcohol (PVA) promptly underwent gelation at room temperature. Macroscopic inversion experiments demonstrated that the gelated Ang‐TEV‐loaded hydrogel (Ang‐TEVs@Gel) manifested excellent mechanical stability, exhibiting no flow or collapse under inverted conditions, which verified its self‐supporting capacity (Figure 2J). Scanning electron microscopy (SEM) was employed to characterize the freeze‐dried Ang‐TEVs@Gel, revealing a uniform and highly interconnected porous structure (Figure 2K). This porous morphology offers sufficient physical space for the efficient encapsulation of extracellular vesicles and enhances their retention through surface interactions.

To evaluate the clinical applicability of the Ang‐TEVs@Gel hydrogel, its injectability was examined using a syringe extrusion experiment (Figure 2L). The results indicated that the hydrogel could be continuously extruded through a 29G needle (outer diameter 0.33 mm) to form uniform filaments, and it rapidly regained its original shape after extrusion. This phenomenon is ascribed to the shear‐thinning behavior of the hydrogel: under high shear stress (during injection), the dynamic phenylboronic ester bonds temporarily rupture, reducing viscosity; once the shear stress is removed, the dynamic bonds quickly reform and restore the gel structure. This characteristic suggests that the hydrogel is suitable for minimally invasive injection and delivery of extracellular vesicles. Fluidity experiments validated the ROS‐responsive behavior, as the phenylboronic ester bonds within Ang‐TEVs@Gel react with hydrogen peroxide to disrupt the hydrogel network and enhance fluidity (Figure 2M). Spinal cord injury initiates pathological cascades such as inflammatory stress, leading to metabolic and functional imbalances in cells and inducing a substantial increase in oxidative stress, which in turn promotes the generation and accumulation of a large quantity of ROS at the injury site. In particular, the dynamic phenylboronic ester bonds in Ang‐TEVs@Gel endow it with ROS‐responsive behavior, enabling the hydrogel to achieve intelligent on‐demand drug delivery. To verify the responsive release performance of Ang‐TEVs@Gel during extracellular vesicle delivery, cumulative release experiments were conducted (Figure 2N). Under physiological conditions (phosphate‐buffered saline, without ROS), the cumulative release of extracellular vesicles within 7 days was merely 27.1% ± 2.5%. However, in a release medium containing 1 mm hydrogen peroxide (simulating a high‐ROS microenvironment), the phenylboronic ester bonds in the Ang‐TEVs@Gel system broke, significantly augmenting the release rate of extracellular vesicles, with a cumulative release of 85.5% ± 0.7% within 7 days. Rheological results indicated that Ang‐TEVs@Gel exhibits mechanical properties similar to native spinal cord tissue, with a storage modulus ranging from 0.1 to 1 kPa [27, 28], supporting its suitability for local implantation (Figure S2A). The swelling ratio of Ang‐TEVs@Gel was approximately 430%, indicating good water absorption capacity (Figure S2B). Under non‐ROS conditions, the degradation profile showed that the hydrogel remained structurally intact with minimal degradation for the first day, degraded about 40% by day 7, and around 70% by day 14 (Figure S2C). Furthermore, we assessed batch‐to‐batch reproducibility for key properties, including rheology, gelation time, swelling ratio, and degradation rate. The results demonstrated consistent performance across different batches, confirming the reliability of Ang‐TEVs@Gel (Figure S2D–G). To assess the retention effect of Ang‐TEVs@Gel in the injured spinal cord, Ang‐TEVs and Ang‐TEVs@Gel were injected into the injury site, and an in vivo imaging system was used to observe the accumulation of Dil‐labeled extracellular vesicles in ex vivo spinal cord tissues on days 1, 4, and 7 (Figure 2O,P). Due to the flushing action of cerebrospinal fluid and blood in the spinal cord, the fluorescence signal of Dil‐labeled EVs in the Ang‐TEVs group rapidly declined on day 1 and was only transiently retained in the spinal cord. In contrast, EVs in Ang‐TEVs@Gel were gradually released from the implantation site into adjacent areas and were still detectable on day 7, indicating that Ang‐TEVs@Gel possesses excellent capability for localized responsive delivery of EVs. To determine whether the hydrogel itself exerts any effect on ROS scavenging, we included a blank hydrogel (Blank‐Gel) control group without Ang‐TEVs in the spinal cord injury model. The results showed that ROS clearance efficacy in the blank hydrogel group was slightly improved compared with the PBS control group, but the difference was not statistically significant (Figure S3A,B). H&E staining results showed that the local delivery of extracellular vesicles to the spinal cord via Ang‐TEVs@Gel did not cause any pathological changes in the major organs (Figure 2Q).

### 2.4 Ang‐TEVs@Gel Enhances Neuromotor Function Recovery After Spinal Cord Injury

**FIGURE 3:**
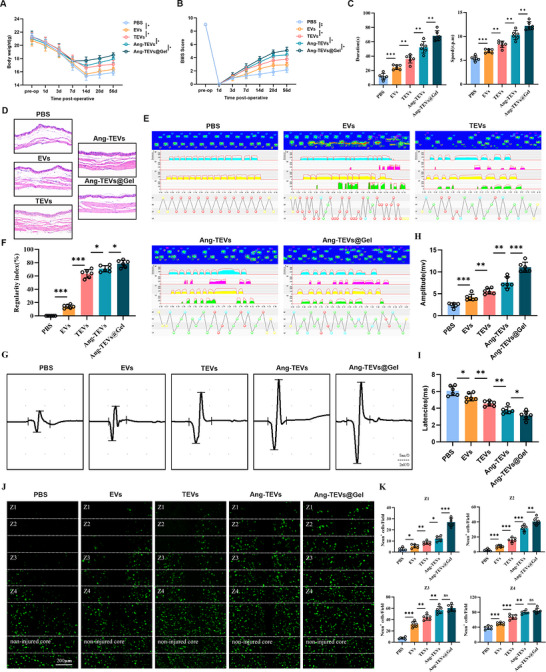
Assessment of neuromotor function recovery in SCI mice following treatment with Ang‐TEVs@Gel. (A) Body weight changes over time in SCI mice treated with PBS, EVs, TEVs, Ang‐TEVs, or Ang‐TEVs@Gel (n = 6). (B) Quantification of locomotor functional recovery using the BMS score over time (n = 6). (C) Statistical evaluation of rotarod test outcomes across five groups (n = 6). (D) Representative H&E staining of bladder tissues across five groups. (E,F) Catwalk and statistical analysis of the five groups (n = 6). (G) Representative waveforms of MEP. (H) Quantitative analysis of MEP amplitude (n = 6). (I) Quantitative analysis of MEP latencies (n = 6). (J,K) Representative immunofluorescence images and quantitative analysis of NeuN‐positive neurons in the Z1‐Z4 regions (n = 6). Non‐injured core: tissue region remote from the epicenter of spinal cord injury. Scale bar, 200 µm.

To evaluate the therapeutic efficacy of Ang‐TEVs@Gel on neuromotor function after spinal cord injury, this study established a mouse model of SCI. The experimental subjects were divided into five groups and treated with PBS, EVs, TEVs, Ang‐TEVs, or Ang‐TEVs@Gel, respectively. By continuously and meticulously monitoring the changes in body weight, the influence of each treatment on the overall condition of mice with SCI was evaluated (Figure 3A). All groups experienced a sharp decline in body weight within 7 days after SCI. The group treated with PBS showed minimal weight restoration, while the group treated with EVs displayed slight signs of weight recovery after the injury. In comparison with the EVs group, treatment with TEVs and Ang‐TEVs further enhanced the overall condition of SCI mice. Moreover, SCI mice treated with Ang‐TEVs@Gel exhibited the most rapid weight recovery among all groups, suggesting the significant protective effect of Ang‐TEVs@Gel against SCI. The Basso Mouse Scale (BMS) scoring results were employed to comprehensively assess the motor function levels of mice in each group (Figure 3B). The PBS‐treated group presented a moderate time‐dependent recovery pattern, whereas the EVs‐treated group demonstrated additional functional improvements, validating the neurorestorative properties of umbilical cord mesenchymal stem cell‐derived EVs. Compared to the EVs group, treatment with TEVs, Ang‐TEVs, and Ang‐TEVs@Gel further promoted the recovery of motor function in SCI mice, with the Ang‐TEVs@Gel treatment group attaining superior therapeutic efficacy.

To comprehensively evaluate the functional recovery of SCI mice, this study conducted multiple functional neuromotor assessments. First, the rotarod test was utilized to evaluate the recovery of motor balance in each group (Figure 3C). The results indicated that mice in the PBS group could only remain on the rotating rod briefly using their forelimbs, while mice in the EVs‐treated group could use their hindlimbs to provide slight assistance. In comparison with the EVs group, mice in the TEVs, Ang‐TEVs, and Ang‐TEVs@Gel treatment groups showed better balance recovery, with the Ang‐TEVs@Gel treatment group effectively maintaining body balance using their hindlimbs. H&E staining of bladder tissues from mice in each treatment group was performed for histological evaluation. The results demonstrated that, compared with the other groups, mice in the Ang‐TEVs@Gel treatment group exhibited markedly restored tissue architecture, indicating that this therapy can effectively alleviate the pathological changes of neurogenic bladder following spinal cord injury (Figure 3D). Subsequently, the CatWalk system was used to evaluate hindlimb gait rhythm and footprint patterns in each group (Figure 3E,F). Mice in the PBS group failed to produce footprints due to joint immobility. Mice in the EVs group exhibited dragging hindlimb footprints, while mice in the TEVs group could land on their dorsiflexed feet but lacked rhythmic hindlimb walking. Mice in the Ang‐TEVs group achieved plantar landing with some recovery of hindlimb gait rhythm, and mice in the Ang‐TEVs@Gel group frequently landed on their hindlimb plantar surfaces with well‐restored gait rhythm. Further electrophysiological analysis of mice in each group revealed that, in contrast to other groups, the Ang‐TEVs@Gel group showed an increase in motor evoked potential (MEP) amplitude and a shortening of latency, indicating improved neural conduction function after treatment with Ang‐TEVs@Gel (Figure 3G,I). To validate the anatomical basis of neurological functional recovery, immunofluorescence staining for the neuronal marker NeuN was performed in different regions (Z1‐Z4) set at varying distances from the injury edge in the injured spinal cord of mice. The results showed that, compared with other treatment groups, the Ang‐TEVs@Gel treatment group exhibited significantly more surviving NeuN‐positive neurons in the Z1‐Z4 regions (Figure 3J,K).

In conclusion, these findings demonstrate that Ang‐TEVs@Gel therapy significantly enhances neuromotor function in mice with SCI.

### 2.5 Ang‐TEVs@Gel Promotes Axonal Regeneration and Neural Circuit Repair by Enhancing Microglial Phagocytosis of Myelin Debris

**FIGURE 4:**
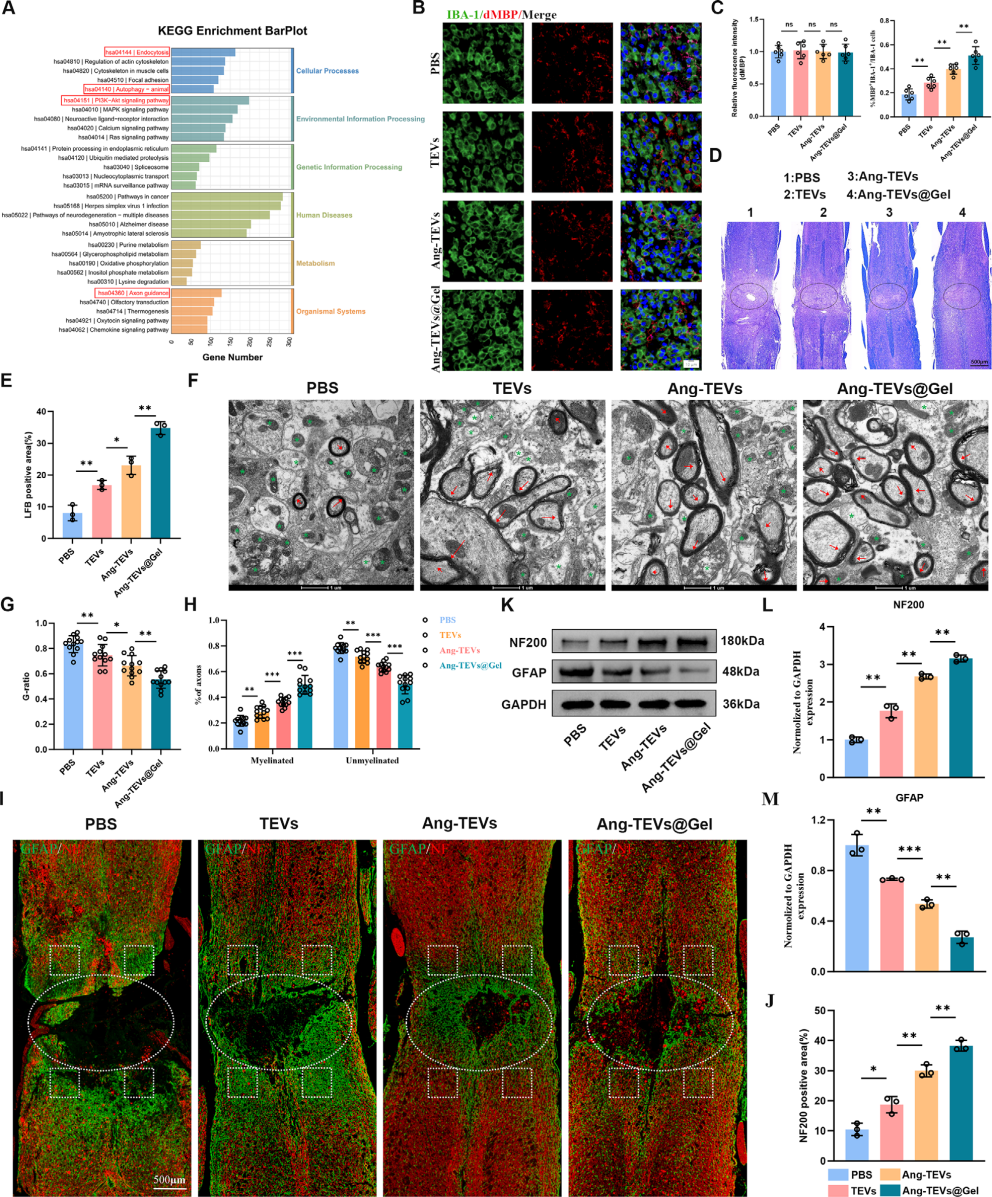
Ang‐TEVs@Gel promotes remyelination and axonal regeneration by enhancing microglial phagocytosis of myelin debris. (A) KEGG pathway enrichment analysis of the target genes of differentially expressed miRNAs differentially expressed between TEVs and EVs. (B) Representative immunofluorescence images showing co‐localization of IBA‐1^+^ microglia (green) and myelin debris (dMBP^+^, red) at 7 days post‐injury. Scale bar, 10 µm. (C) Quantitative analysis of microglial uptake of myelin debris at the spinal cord injury site (n = 6). (D) Representative images of LFB‐stained spinal cord sections. Scale bar, 500 µm. (E) Quantitative analysis of the positively stained myelinated area (n = 3). (F) Representative TEM images of axons in the SCI lesion. Red arrows, myelinated axons; Green asterisks, Unmyelinating axons. Scale bar, 1 µm. (G) Quantitative analysis of the G‐ratio (n = 12). (H) Quantitative analysis of myelinated and unmyelinated axons at the lesion site (n = 12). (I) Representative immunofluorescence images double‐stained for NF‐200 and GFAP. The circles indicate the injury core, and the squares denote the peri‐lesion area (surrounding region adjacent to the injury core). Scale bar, 500 µm. (J) Quantitative analysis of NF200^+^ axon within the lesion area (n = 3). (K–M) Detection of GFAP and NF200 proteins using WB and quantitative analyses (n = 3).

We aim to delve deeper into the intrinsic mechanisms through which Ang‐TEVs@Gel promotes neural functional repair. KEGG enrichment analysis of the target genes of miRNAs differentially expressed between TEVs and EVs indicated that the enhanced intrinsic functions of Ang‐TEVs@Gel are associated with the regulation of pathways related to endocytosis, autophagy, and axonal guidance (Figure 4A). Microglia can promote remyelination by phagocytosing myelin debris, a process critical for neural repair [29, 30]. Based on this, we hypothesize that Ang‐TEVs@Gel facilitates remyelination after spinal cord injury by enhancing microglial phagocytosis of myelin debris.

We assessed the phagocytic activity of microglia for myelin debris in a mouse model of spinal cord injury at 7 days post‐injury. Immunofluorescence staining results showed that compared to PBS, TEVs, and Ang‐TEVs treatment groups, the Ang‐TEVs@Gel group exhibited more significant co‐localization of IBA‐1^+^ microglia and dMBP^+^myelin, indicating enhanced clearance of myelin debris by microglia after Ang‐TEVs@Gel treatment (Figure 4B,C). Luxol fast blue (LFB) staining was used to evaluate post‐injury remyelination levels, revealing that Ang‐TEVs@Gel‐treated mice exhibited robust remyelination capabilities, in stark contrast to the limited therapeutic effects of TEVs and Ang‐TEVs groups and the poorest outcomes in the PBS‐treated group (Figure 4D,E). TEM was employed to observe the ultrastructural morphology of myelin in mice, with quantitative analysis and counting of the G‐ratio for myelinated and demyelinated axons (Figure 4F–H). The PBS group showed extensive demyelination; TEVs treatment partially restored myelin structure, but some vesicular and fragmented myelin remained; the Ang‐TEVs group displayed well‐recovered myelin with clear multilayered structures. Notably, the Ang‐TEVs@Gel group demonstrated the most complete restoration of myelin structure and the highest degree of myelinated axon recovery.

The restoration of myelin structure can facilitate axonal extension, thereby reconstructing neural circuits in the injured area. Immunofluorescence staining with GFAP was used to mark glial scars, while NF200 was employed to visualize spinal cord axons and assess their growth status (Figure 4I,J). In PBS‐treated mice, axonal extension at the injury site was hindered, whereas TEVs and Ang‐TEVs treatments resulted in moderate elongation of nerve fibers. In contrast, Ang‐TEVs@Gel therapy promoted robust axonal growth, with nerve fibers extending through the glial scar and into the lesion core, consistent with the enhanced remyelination and axonal guidance properties of Ang‐TEVs@Gel. Western blot analysis of NF200 and GFAP further validated the trends observed in immunofluorescence staining across the groups (Figure 4K–M).

### 2.6 Ang‐TEVs@Gel Alleviates Secondary Inflammatory Response After Spinal Cord Injury

**FIGURE 5:**
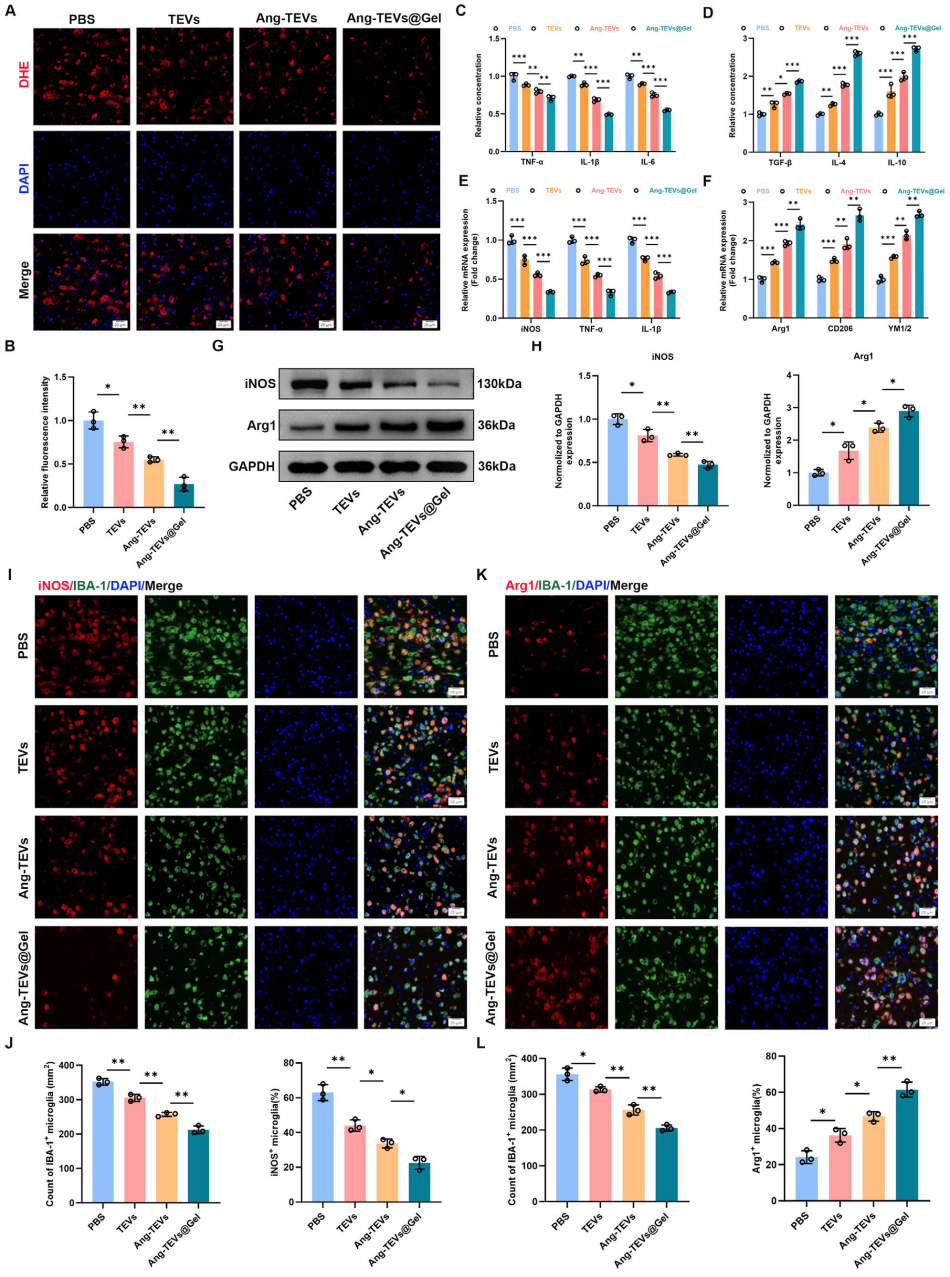
Ang‐TEVs@Gel alleviates the secondary inflammatory response and promotes microglial polarization toward an anti‐inflammatory phenotype after SCI. (A) Representative images of DHE staining showing ROS accumulation (red) in spinal cord sections. Scale bar, 20 µm. (B) Quantitative analysis of DHE fluorescence intensity (n = 3). (C,D) ELISA quantification of pro‐inflammatory cytokines (TNF‐α, IL‐1β, IL‐6) and anti‐inflammatory cytokines (TGF‐β, IL‐4, IL‐10) in spinal cord tissue homogenates (n = 3). (E,F) qRT‐PCR analysis of microglial phenotype‐specific gene expression (n = 3). (G,H) WB and quantitative analysis of proteins corresponding to M1‐ and M2‐associated genes (n = 3). (I,J) Representative immunofluorescence images showing IBA‐1 (green) and iNOS (red) expression and quantitative analyses (n = 3). Scale bar, 20 µm. (K,L) Representative immunofluorescence images displaying IBA‐1 (green) and Arg1 (red) expression, along with corresponding quantitative analyses (n = 3). Scale bar, 20 µm.

Microglia rely on the autophagy pathway to further degrade myelin debris after phagocytosis. Inadequate degradation promotes their shift toward an inflammatory phenotype [31, 32, 33]. We further evaluated the inflammatory levels within the spinal cord microenvironment across diverse treatment groups, while simultaneously measuring the levels of oxidative stress and inflammatory cytokines at the injury site. DHE staining results indicated a notable accumulation of ROS subsequent to spinal cord injury. Treatments with TEVs, Ang‐TEVs, and Ang‐TEVs@Gel progressively reduced oxidative stress levels, with the Ang‐TEVs@Gel treatment exhibiting the most pronounced effect (Figure 5A,B). Subsequently, ELISA was utilized to detect the levels of TNF‐α, IL‐1β, and IL‐6 in the spinal cord. In comparison to the TEVs and Ang‐TEVs treatment groups, the Ang‐TEVs@Gel treatment group significantly diminished the levels of these pro‐inflammatory cytokines. Moreover, the levels of anti‐inflammatory cytokines TGF‐β, IL‐4, and IL‐10 were measured, and their expression patterns were markedly distinct from those of the pro‐inflammatory cytokines (Figure 5C,D).

qRT‐PCR analysis further corroborated that, when compared to the PBS group, the expression of M1‐related genes (iNOS, TNF‐α, and IL‐1β) was downregulated in the TEVs, Ang‐TEVs, and Ang‐TEVs@Gel groups, while the expression of M2‐related genes (Arg1, CD206, and YM1/2) was significantly upregulated. Ang‐TEVs@Gel was more efficacious in promoting the transition of microglia from the M1 to the M2 phenotype (Figure 5E,F). Western blot analysis further validated these findings (Figure 5G,H). Immunofluorescence staining was employed to label iNOS (an M1 marker), Arg1 (an M2 marker), and IBA‐1 (a microglial marker) to assess microglial polarization subsequent to spinal cord injury. In the PBS‐treated group, Arg1 expression was extremely low, whereas iNOS expression was significantly elevated. However, after treatment with TEVs, Ang‐TEVs, and Ang‐TEVs@Gel, Arg1 expression gradually increased, and iNOS expression gradually decreased (Figure 5I–L).

### 2.7 Ang‐TEVs@Gel Regulates the PI3K‐AKT‐mTOR‐Autophagy Pathway in Microglia via miR‐664a‐3p

**FIGURE 6:**
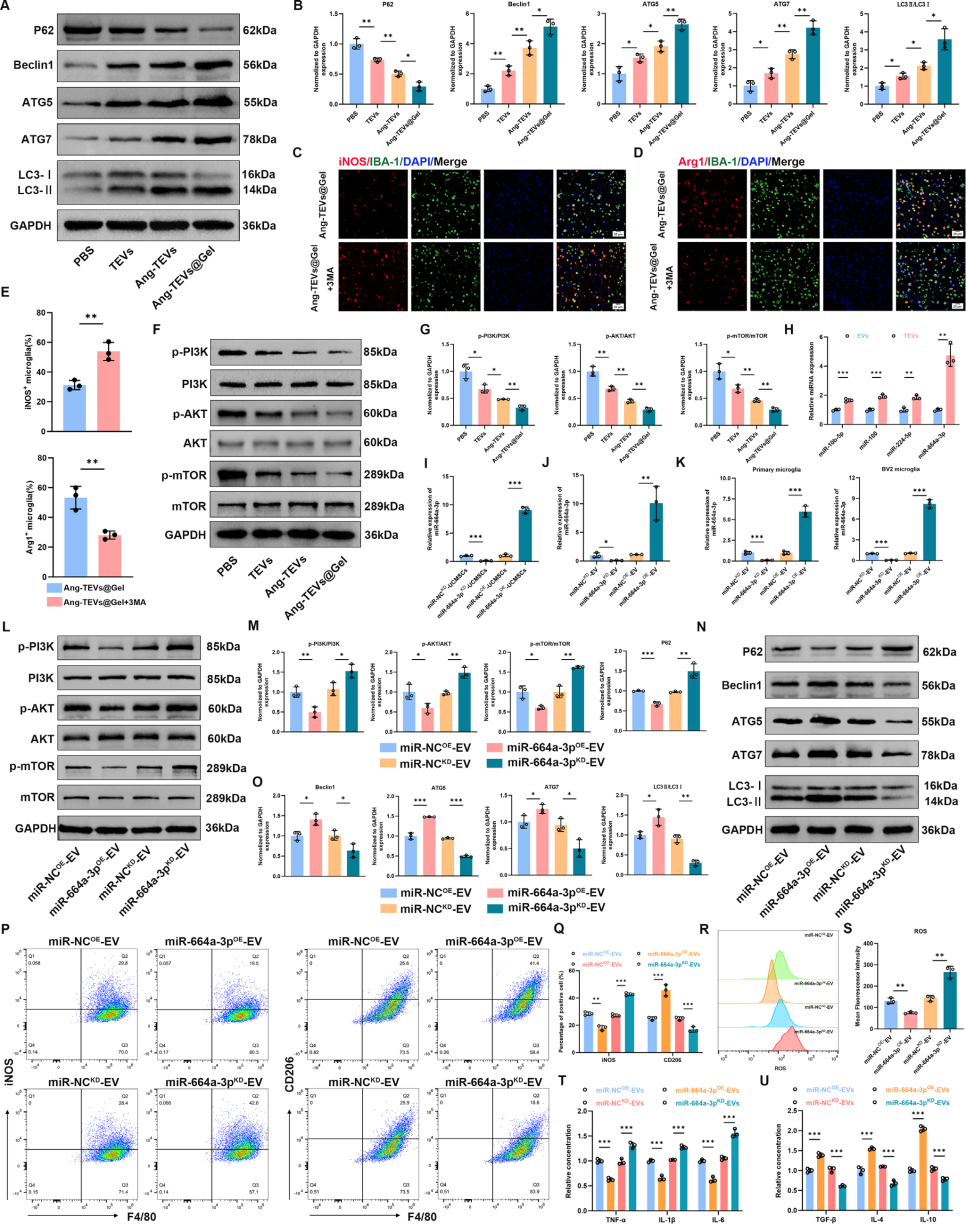
Ang‐TEVs@Gel promotes microglial M2 polarization via miR‐664a‐3p‐mediated inhibition of the PI3K‐AKT‐mTOR pathway to enhance autophagy. (A,B)  Representative Western blots and quantification of key autophagy‐related proteins in spinal cord tissues (n = 3). (C–E) Representative immunofluorescence images showing iNOS or Arg1 expression in microglia and quantitative analyses (n = 3). Scale bar, 20 µm. (F,G)  Representative Western blots and quantification of PI3K‐AKT pathway activation (n = 3). (H) qRT‐PCR detection of miR‐664a‐3p in EVs and TEVs (n = 3).  (I) Transfection efficiency of miR‐664a‐3p overexpression and knockdown in UCMSCs (n = 3).  (J) The relative expression of miR‐664a‐3p in extracellular vesicles derived from the corresponding UCMSCs (n = 3). (K) The relative expression of miR‐664a‐3p in primary microglia and BV2 cells treated with indicated EVs (n = 3). (L–O) miR‐664a‐3p carried by EVs regulates the PI3K‐AKT pathway and autophagy in microglia (n = 3).  (P,Q) Representative flow cytometry plots and quantitative analysis of microglial polarization (n = 3). (R,S) Representative flow cytometry plots and quantitative assessment of ROS levels in microglia (n = 3). (T,U) Quantitative analysis of pro‐ and anti‐inflammatory cytokine expression levels in microglia (n = 3).

Cellular autophagy plays a critical role in regulating microglial degradation of phagocytic substrates and modulating the progression of inflammatory responses following neural injury [32, 34]. Sequencing analysis in this study revealed that differentially expressed miRNAs within Ang‐TEVs@Gel target autophagy‐related pathways (Figure 4A). Accordingly, we hypothesized that Ang‐TEVs@Gel may promote microglial polarization toward the reparative M2 phenotype via autophagy enhancement. Western blot results demonstrated impaired autophagic function post‐spinal cord injury, characterized by elevated accumulation of the autophagy substrate p62 and reduced expression levels of key autophagy proteins Beclin1, ATG5, ATG7, and LC3‐II. Intervention with TEVs, Ang‐TEVs, and Ang‐TEVs@Gel all partially restored autophagic function, with the Ang‐TEVs@Gel group exhibiting the most pronounced effect (Figure 6A,B). Furthermore, compared to Ang‐TEVs@Gel treatment alone, co‐administration with the autophagy inhibitor 3‐methyladenine (3‐MA) significantly increased the M1 phenotype while decreasing the M2 phenotype of microglia. These findings indicate that Ang‐TEVs@Gel modulates microglial polarization through autophagy potentiation (Figure 6C–E). Additionally, sequencing data identified that differentially expressed miRNAs in Ang‐TEVs@Gel target the PI3K‐AKT signaling pathway (Figure 4A), a key negative regulator of autophagy [35]. PI3K‐ AKT pathway activation was observed following spinal cord injury. TEVs, Ang‐TEVs, and Ang‐TEVs@Gel interventions each induced varying degrees of PI3K‐AKT signaling inhibition, with the Ang‐TEVs@Gel group demonstrating the most significant suppression (Figure 6F,G). In summary, Ang‐TEVs@Gel enhances cellular autophagy through inhibition of the PI3K‐AKT signaling pathway, thereby modulating microglial inflammatory responses.

Subsequently, the specific molecular mediator through which Ang‐TEVs@Gel inhibits the PI3K‐AKT signaling pathway was investigated. Differential expression analysis revealed that among the significantly upregulated miRNAs in TEVs compared with EVs, miR‐664a‐3p exhibited the greatest fold change (log_2_FC = 1.73) (Figure 1K), a finding corroborated by qRT‐PCR (Figure 6H). This suggested miR‐664a‐3p as a potential mediator of Ang‐TEVs@Gel‐induced PI3K‐AKT pathway inhibition. To elucidate the functional role of miR‐664a‐3p in modulating the PI3K‐AKT signaling pathway following SCI, lentiviral vector technology was employed to generate UCMSCs with either miR‐664a‐3p overexpression (miR‐664a‐3p^OE^) or knockdown (miR‐664a‐3p^KD^), alongside corresponding negative controls (miR‐NC^OE^ and miR‐NC^KD^). Transfection efficiency was confirmed via qRT‐PCR (Figure 6I). Extracellular vesicles were subsequently isolated from miR‐NC^KD^‐UCMSCs, miR‐664a‐3p^KD^‐UCMSCs, miR‐NC^OE^‐UCMSCs, and miR‐664a‐3p^OE^‐UCMSCs, designated as miR‐NC^KD^‐EVs, miR‐664a‐3p^KD^‐EVs, miR‐NC^OE^‐EVs, and miR‐664a‐3p^OE^‐EVs, respectively. Relative to miR‐NC^KD^‐EVs, miR‐664a‐3p^KD^‐EVs displayed a significant reduction in miR‐664a‐3p expression. Conversely, miR‐664a‐3p^OE^‐EVs exhibited significantly elevated miR‐664a‐3p levels compared with miR‐NC^OE^‐EVs (Figure 6J). Furthermore, microglia treated with miR‐664a‐3p^KD^‐EVs demonstrated significantly lower miR‐664a‐3p expression than those treated with miR‐NC^KD^‐EVs. In contrast, microglia exposed to miR‐664a‐3p^OE^‐EVs showed increased miR‐664a‐3p expression compared with the miR‐NC^OE^‐EVs treatment group (Figure 6K). To simulate an inflammatory milieu, microglia were subsequently stimulated with lipopolysaccharide (LPS). Compared with the miR‐NC^OE^‐EVs treatment group, microglia treated with miR‐664a‐3p^OE^‐EVs exhibited inhibition of the PI3K‐AKT signaling pathway and enhanced autophagic activity. In contrast, relative to the miR‐NC^KD^‐EVs treatment group, the miR‐664a‐3p^KD^‐EVs treatment group displayed significantly enhanced PI3K‐AKT signaling pathway activation and suppressed autophagy (Figure 6L–O). To further substantiate the role of miR‐664a‐3p in promoting autophagic flux in microglia, we performed quantitative analysis of autophagosomes using TEM. The results revealed a significant increase in the number of autophagosomes in microglia treated with miR‐664a‐3p^OE^‐EVs compared with those treated with miR‐NC^OE^‐EVs (Figure S4A,B). We next assessed the integrity of autophagic flux dynamically through a lysosomal inhibition assay. Under steady‐state conditions, the miR‐664a‐3p^OE^‐EVs group exhibited elevated LC3‐II levels and reduced levels of the autophagic substrate p62 relative to the miR‐NC^OE^‐EVs group, indicating enhanced autophagic degradation. Following lysosomal inhibition with bafilomycin A1, the accumulation of both LC3‐II and p62 was significantly greater in the miR‐664a‐3p^OE^‐EVs group than in the miR‐NC^OE^‐EVs group. These findings rule out the possibility of impaired autophagosome–lysosome fusion and confirm that miR‐664a‐3p promotes unimpeded autophagic flux in microglia (Figure S4C,D). Additionally, flow cytometric analysis revealed that, compared with miR‐NC^OE^‐EVs treatment, miR‐664a‐3p^OE^‐EVs treatment reduced M1 polarization while augmenting M2 polarization of microglia. Conversely, miR‐664a‐3p^KD^‐EVs treatment increased M1 polarization and decreased M2 polarization relative to miR‐NC^KD^‐EVs treatment (Figure 6P,Q). Meanwhile, compared to treatment with miR‐NC^OE^‐EVs, administration of miR‐664a‐3p^OE^‐EVs resulted in reduced ROS levels and decreased release of pro‐inflammatory cytokines in microglia, while promoting the secretion of anti‐inflammatory cytokines. Conversely, relative to miR‐NC^KD^‐EV treatment, miR‐664a‐3p^KD^‐EVs led to elevated ROS levels and increased production of pro‐inflammatory cytokines, accompanied by a reduction in anti‐inflammatory cytokine secretion (Figure 6R–U). We further investigated the role of miR‐664a‐3p in modulating microglial polarization in mice with spinal cord injury. The results showed that, compared with the miR‐NC^KD^‐EVs treatment group, the miR‐664a‐3p^KD^‐EVs treatment group exhibited increased M1 polarization and decreased M2 polarization of microglia. Notably, this effect was reversed when miR‐664a‐3p^KD^‐EVs were co‐administered with miR‐664a‐3p mimic (Figure S5A,B). These findings strongly confirm that miR‐664a‐3p is a key effector molecule responsible for the aforementioned therapeutic effects.

In summary, Ang‐TEVs@Gel delivers miR‐664a‐3p to microglia, thereby inhibiting the PI3K‐AKT‐mTOR signaling pathway. This inhibition consequently enhances cellular autophagy and promotes M2 polarization of microglia.

### 2.8 miR‐664a‐3p Targets PIK3CA to Regulate the PI3K‐AKT‐mTOR Pathway in Microglia

**FIGURE 7:**
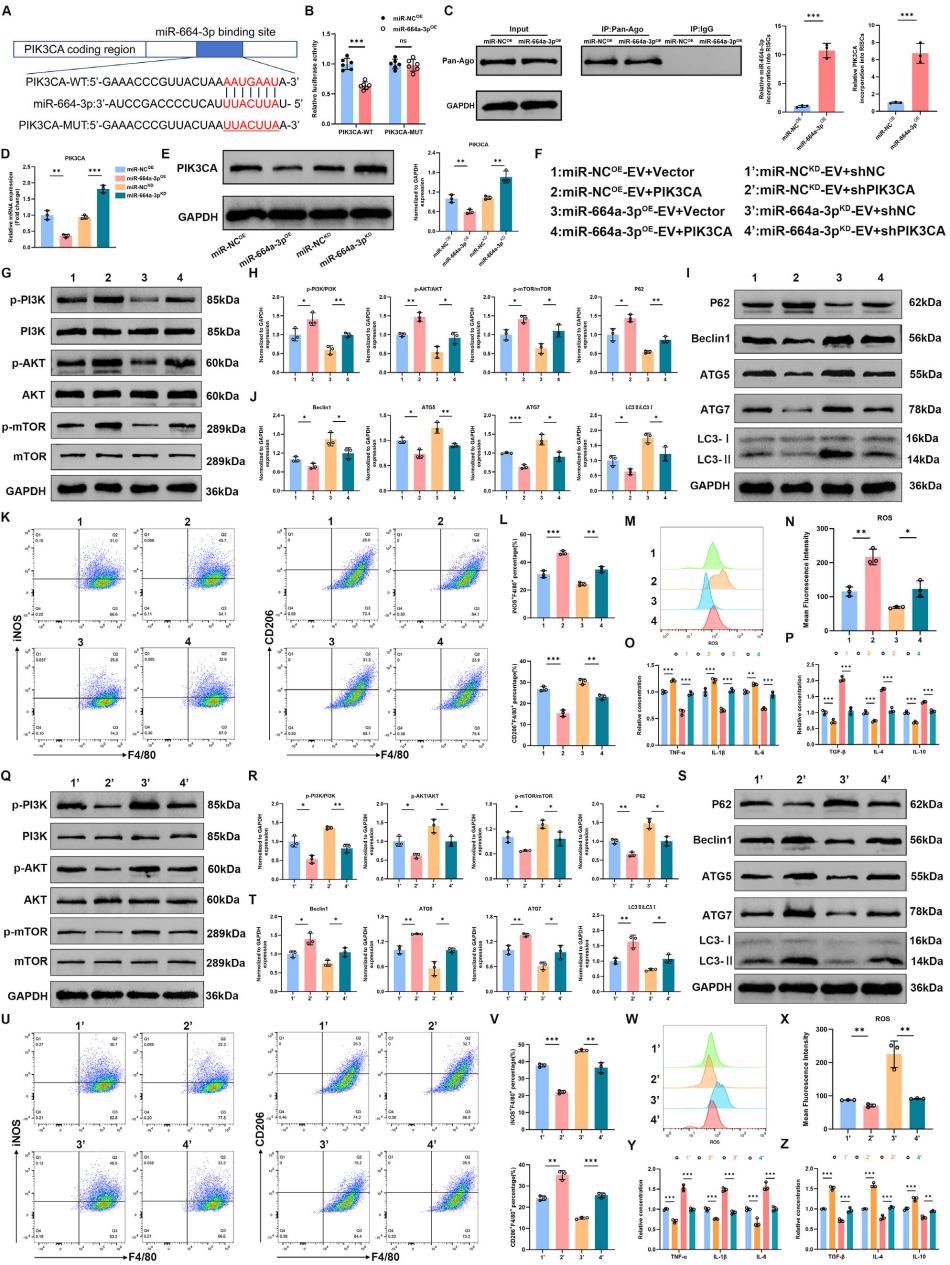
Extracellular vesicle‐derived miR‐664a‐3p inhibits the PI3K‐AKT‐mTOR pathway by directly targeting PIK3CA. (A) Schematic of the binding site for miR‐664a‐3p within the 3′ UTR of PIK3CA. (B) Dual‐luciferase reporter assay verifies the binding of miR‐664a‐3p to PIK3CA (n = 6). (C) RNA‐ChIP analysis. Quantitative PCR showing significant enrichment of PIK3CA mRNA in the Ago2 immunoprecipitate following miR‐664a‐3p overexpression, confirming its loading into the RISC (n = 3). (D,E) qRT‐PCR and Western blot to measure PIK3CA mRNA and protein levels. (F) Schematic overview of the rescue experiment design. (G,H) Representative Western blot images and quantitative analysis of the PI3K‐AKT‐mTOR signaling pathway in the overexpression rescue experiments (n = 3). (I,J) Representative Western blot images and quantitative analysis of autophagy‐related proteins in the overexpression rescue experiments (n = 3). (K,L) Representative flow cytometry analysis and quantitative statistics of microglial polarization phenotypes in the overexpression rescue experiments (n = 3). (M,N) Representative flow cytometry detection and quantitative analysis of ROS levels in microglia in the overexpression rescue experiments (n = 3). (O,P) Detection and quantitative analysis of pro‐inflammatory and anti‐inflammatory cytokine expression levels in microglia in the overexpression rescue experiments (n = 3). (Q,R) Representative Western blot images and quantitative analysis of the PI3K‐AKT‐mTOR signaling pathway in the knockdown rescue experiments (n = 3). (S,T) Representative Western blot images and quantitative analysis of autophagy‐related proteins in the knockdown rescue experiments (n = 3). (U,V) Representative flow cytometry analysis and quantitative statistics of microglial polarization phenotypes in the knockdown rescue experiments (n = 3). (W,X) Representative flow cytometry detection and quantitative analysis of ROS levels in microglia in the knockdown rescue experiments (n = 3). (Y,Z) Detection and quantitative analysis of pro‐inflammatory and anti‐inflammatory cytokine expression levels in microglia in the knockdown rescue experiments (n = 3).

To further elucidate the specific mechanism through which miR‐664a‐3p regulates the PI3K‐AKT‐mTOR signaling pathway, potential target genes of miR‐664a‐3p were screened using the miRDB database and TargetScan database. This analysis identified PIK3CA, encoding the catalytic p110α subunit of the PI3K pathway (Figure 7A). To validate direct targeting of the PIK3CA 3′ UTR by miR‐664a‐3p, wild‐type (WT) and mutant (MUT) PIK3CA 3′ UTR reporter constructs were co‐transfected with miR‐664a‐3p into 293T cells. Dual‐luciferase reporter assays revealed that miR‐664a‐3p overexpression significantly reduced luciferase activity when co‐transfected with the WT PIK3CA 3′ UTR compared with the control group. In contrast, no significant reduction in activity was observed upon co‐transfection with the MUT PIK3CA 3′ UTR (Figure 7B). RNA‐ChIP analysis was performed to quantitatively assess the enrichment of PIK3CA mRNA within the Ago2/RNA‐induced silencing complex (RISC) following miR‐664a‐3p overexpression. Results demonstrated a significant increase in the proportion of PIK3CA mRNA incorporated into the RISC in cells overexpressing miR‐664a‐3p (Figure 7C). Subsequent qRT‐PCR and Western blot analyses confirmed that miR‐664a‐3p knockdown upregulated PIK3CA mRNA and protein expression levels, whereas miR‐664a‐3p overexpression downregulated PIK3CA expression (Figure 7D,E).

To elucidate the functional relationship between extracellular vesicle‐derived miR‐664a‐3p and PIK3CA, rescue experiments were performed (Figure 7F). Lentiviral vectors were used to overexpress PIK3CA in microglial cells. Results indicated that PIK3CA overexpression markedly activated the PI3K‐AKT‐mTOR signaling pathway and reversed the inhibitory effects induced by miR‐664a‐3p^OE^ ‐EVs (Figure 7G,H). Further analysis revealed that PIK3CA overexpression suppressed autophagy (Figure 7I,J), promoted polarization of microglia toward the M1 phenotype (Figure 7K,L), elevated ROS levels (Figure 7M,N), and enhanced the secretion of pro‐inflammatory cytokines (Figure 7O,P). Conversely, endogenous PIK3CA expression was knocked down in microglia using lentivirus‐delivered shRNA. Findings showed that PIK3CA knockdown significantly attenuated PI3K‐AKT‐mTOR pathway activation and reversed the enhancing effects elicited by miR‐664a‐3p^KD^‐EVs (Figure 7Q,R). Moreover, PIK3CA knockdown facilitated autophagy (Figure 7S,T), shifted microglial polarization toward the M2 phenotype (Figure 7U,V), reduced ROS production (Figure 7W,X), and increased the release of anti‐inflammatory cytokines (Figure 7Y,Z). Collectively, these rescue experiments demonstrate that extracellular vesicle‐derived miR‐664a‐3p directly targets PIK3CA, thereby repressing the PI3K‐AKT‐mTOR signaling pathway. This repression leads to enhanced autophagy, a shift in microglial polarization toward a reparative phenotype, reduction in ROS levels, and modulation of inflammatory cytokine secretion.

### 2.9 Verification of the Role of Ang‐TEVs@Gel in Modulating Microglia to Promote Neural Repair Based on snRNA‐seq

**FIGURE 8:**
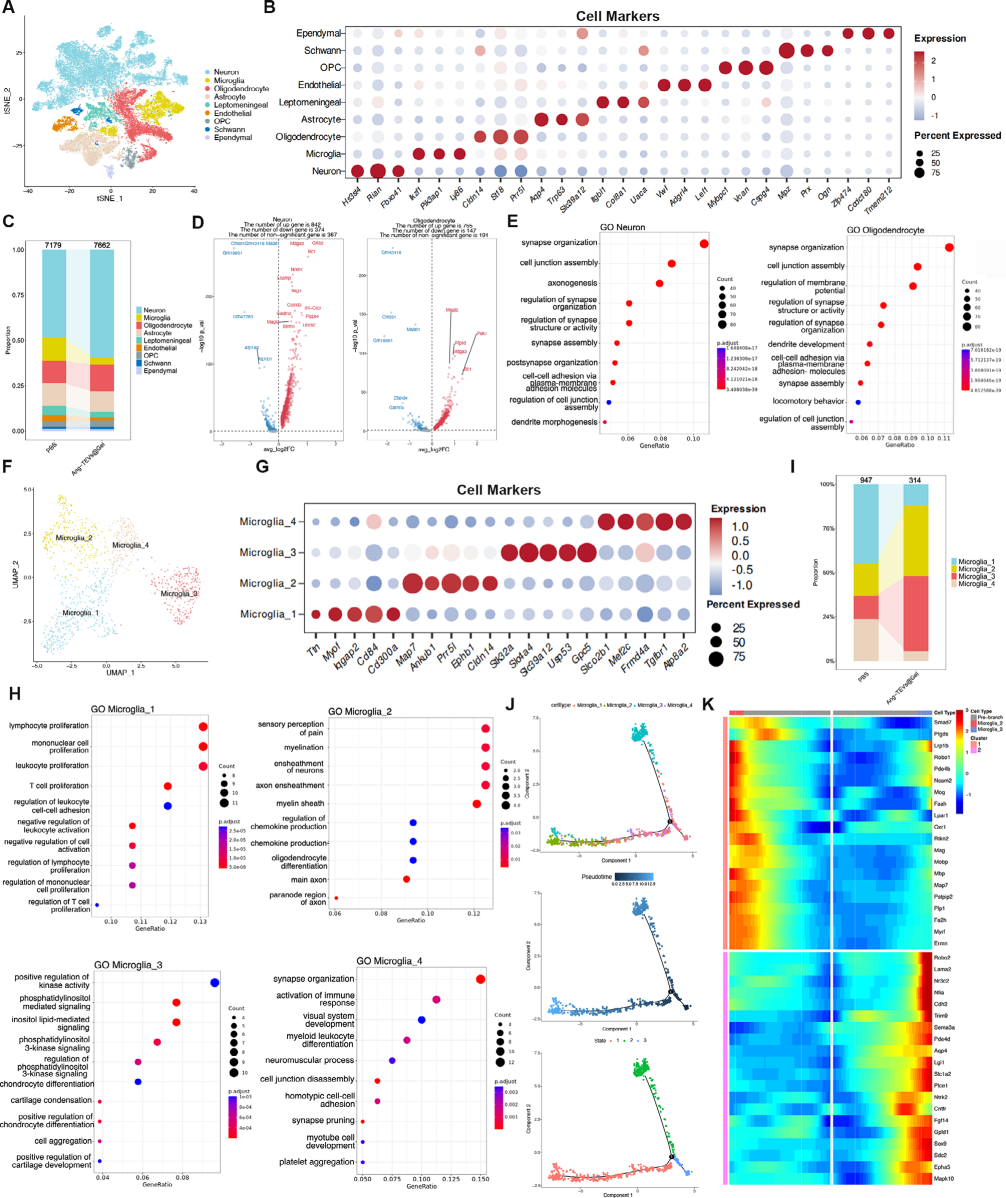
snRNA‐seq analysis reveals that Ang‐TEVs@Gel reprograms microglial subpopulations to promote neural repair. (A) TSNE visualization of all nuclei from PBS‐ and Ang‐TEVs@Gel‐treated spinal cord tissues. (B) Dot plot showing the expression levels of canonical marker genes used for cell type annotation. (C) Stacked bar chart illustrating the proportional changes in cellular composition between PBS and Ang‐TEVs@Gel groups. (D) Volcano plots displaying DEGs in neurons (left) and oligodendrocytes (right) between the two treatment groups. (E) GO enrichment analysis of the biological processes significantly enhanced by Ang‐TEVs@Gel treatment in neurons(left) and oligodendrocytes(right). (F) UMAP plot showing four transcriptionally distinct microglial subsets (Microglia 1–4). (G) Dot plot of representative differentially expressed genes defining each microglial subset. (H) GO functional analysis elucidating the primary biological roles associated with each microglial subset. (I) Proportional composition of the four microglial subsets in PBS vs. Ang‐TEVs@Gel groups. (J) Pseudotime trajectory analysis depicting the dynamic reprogramming of microglial states. (K) Analysis of gene expression dynamics along the pseudotime trajectory.

To validate the efficacy of the aforementioned Ang‐TEVs@Gel on microglial phenotypic transformation following spinal cord injury and its role in neural repair, we conducted single‐nucleus RNA sequencing (snRNA‐seq) on spinal cord tissues at 7 days post‐injury. After rigorous quality control to exclude low‐quality nuclei and potential doublets, we obtained 7,179 nuclei from the PBS‐treated group and 7,662 nuclei from the Ang‐TEVs@Gel‐treated group. These nuclei encompassed nine distinct cell types: neurons, microglia, oligodendrocytes, astrocytes, meningeal cells, endothelial cells, oligodendrocyte precursor cells (OPCs), Schwann cells, and ependymal cells (Figure 8A). Cell type annotation was performed based on significantly differentially expressed genes within each cluster (Figure 8B). Comparative analysis revealed substantial alterations in both cellular composition and gene expression profiles in the Ang‐TEVs@Gel‐treated group, with particularly prominent changes observed in neurons, oligodendrocytes, and microglia (Figure 8C). To further assess the neural repair‐promoting effects of Ang‐TEVs@Gel, differential gene expression analysis was performed separately on neurons and oligodendrocytes. The results demonstrated that, compared to the PBS group, the Ang‐TEVs@Gel group exhibited upregulation of 842 genes and downregulation of 374 genes in neurons, and upregulation of 755 genes and downregulation of 147 genes in oligodendrocytes (Figure 8D). Gene Ontology (GO) enrichment analysis indicated that Ang‐TEVs@Gel treatment significantly enhanced biological processes in neurons associated with synaptic organization, cell junction assembly, and axonogenesis. In oligodendrocytes, it modulated functions related to synaptic organization, regulation of membrane potential, dendritic development, and locomotor behavior (Figure 8E).

To further investigate the influence of Ang‐TEVs@Gel on microglial heterogeneity, microglia from both PBS‐ and Ang‐TEVs@Gel‐treated groups were subdivided into four transcriptionally distinct subsets (Microglia1‐4) for subsequent analysis (Figure 8F,G). GO functional analysis elucidated the specific roles of these microglial subsets: Microglia1 was primarily involved in regulating lymphocyte proliferation, monocyte proliferation, leukocyte proliferation, and T cell proliferation; Microglia2 was mainly associated with myelination, neuron ensheathment, axon ensheathment, and myelin sheath; Microglia3 participated in inositol lipid‐mediated signaling, phosphatidylinositol‐mediated signaling, and phosphatidylinositol 3‐kinase signaling pathways; and Microglia4 primarily contributed to immune response activation and myeloid leukocyte differentiation (Figure 8H). As illustrated in Figure 8I, the PBS‐treated group was predominantly composed of Microglia1 and Microglia4. In contrast, Ang‐TEVs@Gel treatment significantly reduced the proportions of Microglia1 and Microglia4, while markedly increasing the abundances of Microglia2 and Microglia3. Pseudotime trajectory analysis confirmed that Ang‐TEVs@Gel promoted the reprogramming of Microglia1 and Microglia4 toward Microglia2 and Microglia3 (Figure 8J). Analysis of pseudotemporal gene expression changes revealed that during the differentiation of Microglia1 and Microglia4 (Prebranch) toward Microglia2, key neurorepair‐related genes including Mog, Mag, Mobp, Mbp, and Plp1 were upregulated, whereas during differentiation toward Microglia3, inflammation‐suppressing genes such as Robo2, Sema3a, Pde4d, Fgf14, and Trim9 were upregulated (Figure 8K).

In summary, these snRNA‐seq findings are consistent with our earlier experimental results and collectively indicate that Ang‐TEVs@Gel reprograms microglial subpopulations via the PI3K‐AKT signaling pathway, thereby facilitating neural functional recovery after spinal cord injury.

## 3 Discussion

Spinal cord injury constitutes a profoundly debilitating condition, resulting in severe impairment of motor, sensory, and autonomic functions caudal to the injury level. Despite continuous advancements in medical interventions, neurological functional recovery remains limited for many patients, particularly those with complete injuries [36]. This challenge underscores the critical necessity to develop effective therapeutic strategies aimed at promoting neural repair and mitigating secondary complications, including neuroinflammation and oxidative stress. To address the complex pathological cascades following SCI, this study innovatively developed a combined delivery system integrating engineered extracellular vesicles, a ROS‐responsive hydrogel, and targeted immune reprogramming strategies. By synergizing multimodal delivery with immunomodulatory functions, this system is designed to overcome the limitations of existing therapies through enhancing the anti‐inflammatory and neuroregenerative properties of EVs and improving their site‐specific delivery efficiency to the lesion epicenter. Findings derived from this engineered system reveal that Ang‐TEVs@Gel facilitates spatiotemporally controlled EV release and promotes the polarization of microglia toward a pro‐repair phenotype. This effectively attenuates neuroinflammation and ultimately enhances functional recovery post‐SCI. These results provide compelling evidence supporting the development of effective SCI treatments by combining advanced biomaterials with EV‐based therapeutics.

Extracellular vesicles, serving as pivotal mediators of intercellular communication, have been demonstrated to regulate local immunity, promote blood‐spinal cord barrier repair, and enhance axonal regeneration following SCI through the delivery of bioactive components including miRNAs, lncRNAs, and proteins [37]. However, natural EVs exhibit limitations in bioactivity, targeting specificity, and in vivo stability, impeding their clinical translation [38]. Engineered extracellular vesicles refer to EVs modified either by modulating the physiological state of parent cells or by functionalizing the vesicles themselves, thereby addressing the limitations of natural EVs in therapeutic efficacy and delivery efficiency. Current research indicates that for specific disease microenvironments, synergistic optimization of EVs through “functional enhancement” and “targeted delivery” constitutes a critical approach to augmenting their in vivo therapeutic outcomes [39].

Conditionally pretreated extracellular vesicles represent a significant strategy for optimizing the composition and functionality of EV cargoes by regulating the biological state of parent cells. This involves modulating the metabolic status of parent cells via physical, chemical, or biological stimuli (e.g., pharmacological agents, hypoxia, or genetic editing) to directionally alter the miRNAs, proteins, and signaling molecules encapsulated within EVs [40, 41]. For instance, Hu et al. reported that EVs derived from pioglitazone‐pretreated cells significantly enhanced diabetic wound healing [42]. Similarly, Li et al. demonstrated that EVs isolated from tanshinone IIA‐pretreated mesenchymal stem cells improved therapeutic outcomes in myocardial ischemia/reperfusion injury [43]. Our prior investigations further corroborated that EVs secreted by melatonin‐pretreated M2 microglia exhibited superior efficacy in spinal cord injury repair [21]. In the present study, TMP pretreatment of umbilical cord mesenchymal stem cells altered the profile of functional miRNAs within their EVs, consequently enhancing anti‐inflammatory and pro‐axonal regeneration capacities. Nevertheless, given that EVs encapsulate diverse bioactive components (e.g., proteins, lncRNAs), the potential contribution of other cargoes to these functional enhancements cannot be excluded.

Targeted modification constitutes a pivotal research domain within the engineering of EVs. Native EVs inherently lack targeting specificity, rendering them subject to rapid systemic clearance and non‐specific biodistribution across bodily tissues. This limitation results in suboptimal therapeutic concentrations at target sites and an elevated risk of off‐target adverse effects. Conjugation of specific ligands onto the EV surface enables precise recognition of molecules highly expressed within intended pathological microenvironments, thereby substantially enhancing their site‐specific retention and enrichment efficiency [44, 45, 46]. For instance, Rong et al. enhanced EV targeting to SCI sites via conjugation of CAQK peptides [47]. Similarly, Xie et al. improved EV targeting to pathological vasculature within injured regions using RGD peptides [48]. Our prior research also demonstrated the efficacy of Ang2 peptide modification in augmenting EV targeting specificity toward microglia post‐SCI [21, 26]. In the current study, Ang‐TEVs further potentiated their targeted penetration into microglia within the injured spinal cord region through Ang2‐LRP1‐mediated active targeting. This modification significantly amplified their capacity to modulate neuronal function and remodel the local immune microenvironment. Furthermore, targeted modification facilitates the flexible selection and adjustment of ligand types tailored to diverse disease targets, enabling prospects for personalized therapeutic strategies and representing a key technology driving the clinical translation of engineered EVs. However, despite intravenous administration of targeted engineered EVs increasing their accumulation within the SCI area, significant hepatic sequestration persisted. Therefore, we subsequently implemented hydrogel‐based local delivery for in situ treatment of the injury.

The therapeutic application of hydrogel‐encapsulated EVs in SCI management has been extensively documented [23, 49]. For example, Fan et al. developed an electroconductive hydrogel incorporating EVs that facilitates post‐SCI tissue regeneration through synergistic immunomodulation and enhanced myelinated axon growth [50]. Wang et al. engineered an injectable, self‐healing anti‐inflammatory bioactive hydrogel exhibiting ultra‐sustained EV release kinetics, which collectively improved functional motor recovery following SCI [51]. Cao et al. designed an augmented EV‐integrated hydrogel featuring a ROS‐responsive release mechanism, effectively mitigating pathological progression in SCI [52]. Nevertheless, these approaches encounter persistent translational challenges, including uncontrolled EV release kinetics, absence of lesion‐specific targeting, and insufficient inherent EV bioactivity. Compared to existing material systems, the hydrogel platform developed in this study demonstrates distinct multidimensional advantages. This hydrogel specifically responds to elevated ROS concentrations within the SCI lesion microenvironment, enabling precise, on‐demand EV release that overcomes uncontrolled delivery limitations. Its superior biocompatibility and biomechanical compatibility ensure seamless integration with host tissues, not only guaranteeing prolonged EV release stability but also substantially reducing immunogenic rejection risks. The hydrogel's three‐dimensional network architecture provides essential structural scaffolding to local tissues, establishing a conducive microenvironment for myelin regeneration and neural repair. Notably, building upon pre‐treatment enhancement of intrinsic EV bioactivity, this study integrates engineered Ang2 peptide‐modified EVs with a ROS‐responsive hydrogel. This strategy achieves both localized responsive sustained EV release and post‐release active targeting functionality. Consequently, it significantly enhances Ang‐TEVs’ cellular affinity and therapeutic specificity toward injured neural substrates, thereby comprehensively improving the efficacy and reliability of this therapeutic paradigm.

Demyelination constitutes a key pathological mechanism underlying secondary injury following SCI. The status of the local microenvironment subsequent to myelin debris clearance critically influences the efficiency of remyelination [53]. Microglia, serving as the predominant resident immune cells within the spinal cord, play a pivotal role in this process by recognizing and phagocytosing degenerated myelin debris via surface receptors. The phagocytosed myelin debris relies upon the cellular autophagy pathway for effective degradation. Impairment of autophagy or incomplete degradation leads to the accumulation of lipotoxic metabolites, driving microglia toward a pro‐inflammatory phenotype. This shift results in the release of inflammatory factors, such as TNF‐α and IL‐1β, thereby establishing an inhibitory microenvironment for regeneration and ultimately compromising myelin repair [31, 32, 33, 34]. Consequently, modulating the phagocytic and autophagic functions of microglia represents a crucial potential therapeutic strategy for promoting spinal cord repair. In the present study, pretreatment with TMP significantly enhanced the ability of EVs to promote cellular phagocytosis and autophagy. Subsequently, Ang2 peptide was utilized to achieve targeted delivery of EVs to microglia. This strategy markedly enhanced microglial phagocytosis of myelin debris while simultaneously augmenting their autophagic degradation capacity. Consequently, it promoted the transition of microglia toward a reparative phenotype, suppressed the formation of pro‐inflammatory microglia, and effectively improved the post‐SCI microenvironment. Thus, microglial immune reprogramming not only optimized their myelin debris clearance function but also indirectly mitigated inflammation, establishing a positive feedback loop from immune regulation to tissue repair. This synergistic regulatory strategy also significantly enhanced the capacity of EVs to adapt to complex pathological environments.

The PI3K‐AKT signaling pathway functions as a pivotal upstream regulator of cellular autophagy. Activated AKT phosphorylates and inhibits TSC2, consequently activating mammalian target of rapamycin complex 1 (mTORC1). mTORC1 constitutes a key negative regulator of autophagy; it suppresses autophagosome formation by phosphorylating components of the autophagy initiation complex, namely ULK1/2 and ATG13 [35, 54]. Substantial evidence indicates that pharmacological or genetic inhibition of the PI3K/AKT/mTOR pathway induces autophagic activity. For instance, cadmium exposure accelerates autophagy in osteocytes by suppressing PI3K/AKT/mTOR signaling [55]. Similarly, magnolol induces cytotoxic autophagy in glioma models through PI3K/AKT/mTOR pathway inhibition [56]. Conversely, eupahiol inhibits lipopolysaccharide‐induced cellular autophagy by activating the PI3K/AKT/mTOR signaling pathway [57]. These collective findings demonstrate the PI3K‐AKT‐mTOR axis as a core regulatory hub constraining autophagic flux. In the present study, TMP pretreatment significantly potentiated the inhibitory effect of EVs on the PI3K‐AKT‐mTOR axis, thereby enhancing microglial autophagy and promoting polarization toward the M2 phenotype. EVs function as efficient intercellular communicators, transmitting diverse cargo components–‐including proteins, lipids, and nucleic acids such as miRNAs–‐to recipient cells, thereby modulating key signaling pathways including PI3K‐AKT [58]. For instance, small EVs derived from human exfoliated deciduous teeth stem cells are enriched with miR‐200c‐3p, which activates PI3K/AKT signaling by targeting PTEN, a negative regulator of this pathway [59]. Analogously, M2 macrophage‐derived EVs deliver miR‐222‐3p, activating the downstream PI3K/AKT/mTOR pathway through TSC1 inhibition [60]. These studies collectively indicate that EVs serve as efficient delivery systems for PI3K‐AKT pathway‐modulating molecules, with their specific cargo composition dictating the regulatory outcome on recipient cell signaling and functional fate. Our investigation revealed that TMP pretreatment significantly elevated miR‐664a‐3p content within EVs. This miRNA targets the PIK3CA gene (encoding p110α, a catalytic subunit essential for PI3K pathway activation), thereby inhibiting the PI3K‐AKT‐mTOR axis and promoting cellular autophagy. Elucidation of this novel regulatory mechanism may inform future therapeutic strategies targeting neuroinflammation and neurodegeneration following spinal cord injury.

This study provides compelling evidence for the application of Ang‐TEVs@Gel in spinal cord injury treatment, yet several limitations remain to be addressed in future research. The therapeutic contributions of other bioactive components within TEVs are still unclear. Although miR‐664a‐3p has been confirmed as the core molecule mediating the effects of TEVs, EVs simultaneously carry various active substances such as proteins, lncRNAs, circRNAs, and lipids, and the synergistic or auxiliary contributions of these components to the overall therapeutic efficacy require systematic elucidation. The upstream sorting mechanism by which TMP pretreatment promotes the selective enrichment of miR‐664a‐3p in EVs also awaits clarification. Whether RNA‐binding proteins (e.g., hnRNPA2B1, YBX1) participate in the TMP‐regulated cargo sorting process and their specific signaling pathways need further in‐depth investigation. Furthermore, the targeting efficiency and functional impact of Ang‐TEVs on astrocytes, endothelial cells, and other LRP1‐expressing cells within the injured microenvironment currently lack systematic quantitative assessment; single‐cell resolution uptake mapping would facilitate a comprehensive evaluation of their targeting specificity. Regarding model validation, this study only observed therapeutic efficacy in a mouse contusion model, and translation to human SCI is limited by interspecies differences in spinal cord anatomy, inflammatory response dynamics, and regenerative capacity. Long‐term safety and efficacy require further evaluation in large animal models. At the sequencing technology level, the limited depth of snRNA‐seq failed to adequately capture known functional subpopulations such as disease‐associated microglia (DAM) and proliferation‐associated microglia (PAM); deeper sequencing and integration of spatial transcriptomics would help reveal the comprehensive regulation of microglial heterogeneity by Ang‐TEVs@Gel. Finally, the multi‐step engineering process and batch‐to‐batch variability of EV preparations pose challenges for scale‐up production and quality control in clinical translation. Establishing standardized manufacturing processes is an essential step toward clinical application.

In summary, the engineered EVs‐hydrogel combination delivery system developed in this study achieved spatiotemporal synergistic regulation, enabling precise targeted delivery of extracellular vesicles, microglial immune reprogramming, clearance of myelin debris, and promotion of remyelination for spinal cord injury treatment. Through multi‐target cascade regulation, this system overcomes the limitations inherent in conventional single‐strategy approaches, thereby establishing both theoretical and practical foundations for comprehensive SCI intervention.

## 4 Materials and Methods

### 4.1 Cell Culture

hUCMSCs were commercially acquired from Cyagen Biosciences Inc. (Cat. No. HUXUC‐01001). Cell cultures were maintained under sterile conditions in complete medium for hUCMSCs (Cat. No. HUXUC‐90011) within a humidified incubator at 37°C with 5% CO_2_saturation. Medium replenishment was performed approximately every 2–3 days. Upon reaching 80%–90% confluence, cells were subjected to enzymatic dissociation using 0.25% trypsin and subsequently passaged at a 1:3 ratio into fresh culture vessels. Cells from passages 3 to 5 were utilized for all experimental procedures. For the preparation of TEVs, the original medium was aspirated when cell confluence attained approximately 70%. Cultures were gently rinsed twice with PBS. The medium was then replaced with complete medium supplemented with TMP. Parallel control cultures (employed for conventional EV preparation) received an equivalent volume of standard complete medium. Following 24 h of continued incubation under pretreatment conditions, subsequent vesicle isolation procedures were initiated.

### 4.2 Flow Cytometric Phenotypic Characterization

hUCMSCs in the logarithmic growth phase were harvested and digested using trypsin. The resulting cell suspension was resuspended in PBS supplemented with 2% FBS (designated as FACS buffer), and the cell density was adjusted to 1 × 10^6^ cells/mL. Aliquots of 100 µL cell suspension were dispensed into flow cytometry tubes. Monoclonal antibodies targeting CD90, CD105, CD73, CD45, and CD34 (Product No. HUXMX‐09011; Cyagen Biosciences Inc.) were added simultaneously. Antibody incubation was performed at 4°C in the dark for 30 min. Subsequently, FITC‐conjugated secondary antibodies were introduced, and the reaction proceeded in the dark for an additional 20 min. Following each staining step, cells were washed twice with FACS buffer (centrifugation at 350 × g for 5 min per wash). After the final wash, cells were resuspended in 300 µL of PBS. Flow cytometric data acquisition was conducted using an isotype control antibody as a negative control. All data analyses were performed using FlowJo software (version 10.8.1).

### 4.3 Extraction and Basic Characterization of Extracellular Vesicles

The supernatant was transferred to ultracentrifugation tubes (Beckman). Sequential centrifugation was performed at 300 × g (10 min), 2000 × g (20 min), and 10 000 × g (30 min) to remove cellular debris, followed by final centrifugation at 100 000 × g (70 min, Beckman) to collect extracellular vesicles, and the final pellet was resuspended in 100 µL of PBS. To avoid absolute concentration bias arising from the choice of resuspension volume, this study ensured biological comparability across different dosing regimens in both in vitro and in vivo functional experiments by fixing the initial cell culture number and strictly controlling the final resuspension volume. The resultant pellet was resuspended in PBS and stored at −80°C. Extracellular vesicle size distribution was measured using DLS with a Malvern particle size analyzer. For TEM, a 10 µL aliquot of the EV suspension was applied to a carbon‐coated copper grid and incubated at room temperature for 5 min. Excess liquid was removed using filter paper. Subsequently, 10 µL of 2% phosphotungstic acid solution was applied for negative staining for 1 min. The staining solution was removed using filter paper, and the grid was air‐dried overnight at room temperature. Samples were imaged using a transmission electron microscope.

### 4.4 Western Blot Analysis

Protein extraction was conducted using RIPA lysis buffer. Subsequently, protein concentration was quantified via the BCA assay to ensure accurate subsequent analysis. Proteins were resolved by molecular weight employing 12.5% SDS‐PAGE and electrophoretically transferred onto a PVDF membrane. The membrane was blocked with 5% BSA for 1 h at room temperature. Following blocking, the membrane was incubated overnight at 4°C with target‐specific primary antibodies. Post‐incubation, the membrane underwent three washes with 1% TBST to remove unbound primary antibodies, followed by a 1‐hour incubation at room temperature with corresponding HRP‐conjugated secondary antibodies to amplify detection signals. Protein bands were visualized using ECL reagent to ascertain band position and relative intensity. The antibodies utilized in this study were: anti‐CD9 (Proteintech Group, Inc., 1:2000), anti‐CD63 (Abcam plc, 1:1000), anti‐CD81 (Proteintech Group, Inc., 1:2000), anti‐Alix (Proteintech Group, Inc., 1:5000), anti‐TSG101 (Proteintech Group, Inc., 1:1000), anti‐Calnexin (Proteintech Group, Inc., 1:5000), anti‐iNOS (Proteintech Group, Inc., 1:2000), anti‐Arg1 (Proteintech Group, Inc., 1:5000), anti‐GFAP (Abcam plc, 1:1000), anti‐NF200 (Proteintech Group, 1:5000), anti‐GAPDH (Proteintech Group, Inc., 1:5000), anti‐P62 (Proteintech Group, Inc., 1:2000), anti‐Beclin1 (Proteintech Group, Inc., 1:2000), anti‐ATG5 (Proteintech Group, Inc., 1:2000), anti‐ATG7 (Proteintech Group, Inc., 1:2000), anti‐LC3B (Abcam plc, 1:2000), anti‐p‐PI3K (CST, Inc., 1:1000), anti‐PI3K(Abcam plc, 1:2000), anti‐p‐AKT (Proteintech Group, Inc., 1:2000), anti‐AKT (Proteintech Group, Inc., 1:2000), anti‐p‐mTOR (Proteintech Group, Inc., 1:2000), anti‐mTOR (Proteintech Group, Inc., 1:1000).

### 4.5 Preparation of Targeted Peptide‐Modified Vesicles (Ang‐TEVs)

Ang‐TEVs were prepared via a two‐step click chemistry procedure. The initial step entailed DBCO activation: purified extracellular vesicles were incubated with DBCO‐sulfo‐NHS under constant rotation at room temperature for 4 h. Subsequently, three sequential centrifugal washes were performed using a 100 kDa molecular weight cut‐off ultrafiltration centrifuge tube (Millipore) to completely remove unreacted DBCO reagents. Following activation, peptide conjugation was conducted: the DBCO‐functionalized TEVs were reacted with azide‐modified Angiopep‐2 peptide or Cy5.5 dye in a rotary shaker at 4°C for 12 h. Similarly, three washing cycles employing the 100 kDa ultrafiltration centrifuge tube were executed to eliminate unconjugated peptides and dyes. The final modified extracellular vesicle products were obtained by resuspension.

### 4.6 Preparation and Characterization of Ang‐TEVs@Gel

Hydrogels were synthesized via boron ester dynamic bonding. Briefly, HA‐PBA (Ruixi, Xian, China) at a concentration of 2.5 wt.% and PVA (Macklin, Shanghai, China) at a concentration of 2.5 wt.% were prepared in sterile PBS to form homogeneous mixtures, respectively. Then, these mixtures were combined in a volume ratio of 3:1 at room temperature to create HA‐PBA/PVA hydrogels. The engineered exosomes (Ang‐TEVs) were dispersed in the PVA solution to prepare the hydrogel named Ang‐TEVs@Gel.

The samples were secured onto a metal base and sprayed with gold under vacuum. Then, the microstructures of the samples were visually observed by field‐emission scanning electron microscopy.

### 4.7 EVs Release of Ang‐TEVs@Gel

Ang‐TEVs@Gel was placed in 5 mL of PBS solution or H_2_O_2_ (1 mm) solution. At 0, 1, 2, 4, 8, 16, 24, 36, 48, 72, 96, 120, 144, and 168 h, 200 µL of solution was collected, and 200 µL of fresh PBS or H_2_O_2_ solution was added. The release amount of EVs was measured using a microBCA assay kit. Fresh H_2_O_2_ was added daily to maintain its concentration. Hydrogel degradation was observed daily.

### 4.8 Establishment of Mouse Spinal Cord Contusion Model

All animal experimental procedures were approved in accordance with the guidelines of the Animal Ethics Committee of Nanjing Medical University (Approval No.: 2022‐SRFA‐436). Eight‐ to ten‐week‐old male C57BL/6J mice, weighing 20—22 g body weight, and housed under SPF conditions, were utilized in this study. Experimental mice were anesthetized via intraperitoneal injection of sodium pentobarbital. Following anesthesia, the dorsal surgical site was shaved and disinfected aseptically. A midline dorsal skin incision was made, the paravertebral muscles were gently dissected, and a laminectomy was performed at the T9 vertebral level to fully expose the underlying spinal dura mater. A spinal cord contusion injury was subsequently established at the T9/T10 spinal level using the Louisville Injury System Apparatus (LISA). Muscle fasciae and skin incisions were sutured in layers. Postoperatively, manual bladder expression was performed twice daily until spontaneous micturition function recovered.

### 4.9 In Vivo Imaging and Biodistribution

To assess targeting efficacy, a cohort of mice underwent in vivo imaging studies. Cy5.5‐labeled EVs, TEVs, or Ang‐TEVs (100 µg in 100 µL PBS) were administered intravenously via tail vein injection. Whole‐body imaging was performed at predetermined time points post‐injection using an IVIS in vivo imaging system. Following anesthesia induction with isoflurane, fluorescence signals within the dorsal spinal cord region were captured. To evaluate the local retention kinetics of the hydrogel, Dil‐labeled Ang‐TEVs or an equivalent dose of Ang‐TEV‐loaded hydrogel (Ang‐TEVs@Gel) was administered via direct injection into the lesion site. Animals were euthanized on postoperative days 1, 4, and 7, followed by extraction of the entire spinal cord column. Ex vivo fluorescence imaging of spinal cord tissues was conducted using the IVIS platform, with quantitative analysis of fluorescence intensity within the injured region of interest (ROI) performed utilizing ImageJ software.

### 4.10 Behavioral Assessment

BMS assessments were performed on postoperative days (POD) 1, 3, 7, 14, 28, and 56. Two blinded investigators independently evaluated hindlimb motor function within an open‐field arena, assigning scores according to the established 0–9 point scale. The rotarod test was conducted on POD 56. Mice were positioned on the rotarod apparatus operating in constant acceleration mode; the latency to fall and corresponding rotational speed were recorded. Each animal underwent three trials, with the longest latency period utilized for analysis. CatWalk XT automated gait analysis was executed on POD 56. Mice were permitted to traverse freely along an enclosed glass walkway, during which a high‐speed camera system automatically captured, recorded footprint images, and analyzed gait parameters. Motor Evoked Potential (MEP) recordings were obtained 56 days post‐injury. The stimulating electrode was positioned on the exposed ventral spinal cord; the recording electrode was inserted into the biceps femoris flexor muscle, the reference electrode was affixed to the distal tendon of the hindlimb, and the ground electrode was placed subcutaneously. MEP responses were elicited using single‐pulse stimulation (10 mA, 0.1 ms duration, 1 Hz frequency) and measured.

### 4.11 Immunofluorescence Staining

Following cardiac perfusion, mouse spinal cord tissue samples were promptly harvested and fixed in 4% paraformaldehyde to preserve tissue architecture, minimize autolysis, and prevent protein degradation. Post‐fixation, tissues underwent sequential dehydration, paraffin embedding, and sectioning. Sections were incubated with blocking buffer to mitigate non‐specific antibody binding. Subsequently, sections were incubated overnight at 4°C with target‐specific primary antibodies to ensure optimal antigen‐antibody interaction. The following day, sections were incubated with fluorophore‐conjugated secondary antibodies for 1 h at ambient temperature. To facilitate nuclear identification during microscopic analysis, sections were counterstained with DAPI.

The following primary antibodies were employed in this study: Anti‐NF200 antibody (Abcam, 1:1000) for neurofilament detection; Anti‐NeuN antibody (Abcam, 1:3000) as a neuronal marker; Anti‐IBA‐1 antibody (Abcam, 1:2000) for microglial identification; Anti‐iNOS antibody (Abcam, 1:5000) as an M1 microglial polarization marker; Anti‐Arg1 antibody (Proteintech, 1:3000) as an M2 microglial polarization marker.

### 4.12 Histochemical Staining

#### 4.12.1 LFB Staining

This technique was employed to evaluate myelin integrity. Tissue sections were immersed in LFB staining solution overnight at 56°C, protected from light. Subsequent differentiation was performed sequentially in lithium carbonate solution and 70% ethanol. Following dehydration, clearing, and mounting, sections were examined and imaged using light microscopy. The percentage of the blue‐stained area within the lesion region was quantified for subsequent analysis.

#### 4.12.2 TEM Sample Preparation

Tissue blocks (∼1 mm^3^) were dissected from the epicenter of the injury site. Samples underwent sequential fixation in 2.5% glutaraldehyde (in 0.1 m phosphate buffer) and 1% osmium tetroxide, followed by dehydration through a graded ethanol series. Tissues were then embedded in Epon epoxy resin. Ultrathin sections (70 nm thickness) were prepared, stained with uranyl acetate and lead citrate, and subsequently examined and imaged using a transmission electron microscope. The G‐ratio, (defined as the ratio of the inner axonal diameter to the total outer diameter of the myelinated fiber, was measured in random fields using ImageJ software.

### 4.13 ELISA

Cell culture supernatant or tissue homogenate samples were collected, centrifuged to remove particulate matter, and subsequently subjected to analysis. Standards and test samples were aliquoted into wells of an ELISA plate pre‐coated with a capture antibody. A standard curve was generated using serial dilutions of the reference standard. The plate was incubated at 37°C for a defined period to facilitate antigen‐antibody binding. Following incubation, the liquid contents were aspirated, and wells were washed five times with wash buffer to eliminate unbound components and minimize non‐specific background. An enzyme‐conjugated detection antibody was then added, and the plate underwent a secondary incubation at 37°C to facilitate specific binding. Post‐incubation, the liquid was discarded and the washing procedure was repeated. Substrate solution was introduced to initiate the enzyme‐catalyzed chromogenic reaction. The reaction was terminated by the addition of stop solution, and the optical density (OD) was quantified at the specified wavelength using a microplate reader. The cytokine concentration in each sample was determined by interpolating the measured OD values against the established standard curve.

### 4.14 Real‐Time Quantitative PCR

Total RNA was isolated from cells or extracellular vesicles using TRIzol reagent (Invitrogen, USA) to serve as template for cDNA synthesis. cDNA synthesis for miRNA analysis was performed using the Hairpin‐it miRNA qPCR Quantitation Kit (GenePharma, China). Conversely, cDNA synthesis for mRNA analysis was carried out using the PrimeScript RT Kit (Takara, Japan). Subsequently, qRT‐PCR analysis was performed using the TB Green Premix Ex Taq Kit (Takara, Japan) to quantify mRNA and miRNA expression levels. To ensure data accuracy, relative expression levels were calculated using the established 2^(‐ΔΔCt) method, normalized against the internal reference genes GAPDH for mRNA and U6 for miRNA, respectively.

### 4.15 Dual‐Luciferase Reporter Assay

Sequences encompassing either the wild‐type (WT) or mutant (MUT) fragment of the PIK3CA 3′UTR were synthesized, followed by cloning into luciferase reporter vectors. The resultant reporter plasmids were co‐transfected into HEK293T cells in combination with either a miR‐664a‐3p mimic or a negative control mimic. Luciferase activity was quantified utilizing a dual‐luciferase reporter assay system 48 h post‐transfection.

### 4.16 RNA Immunoprecipitation

Cells were initially fixed with 1% formaldehyde to induce protein‐nucleic acid cross‐linking. Subsequently, the cross‐linked chromatin was subjected to sonication to generate fragments suitable for immunoprecipitation. Cellular lysis was performed using an appropriate buffer to ensure complete release of intracellular components. Immunoprecipitation was conducted utilizing the Dynabeads Protein A magnetic bead system (Invitrogen). Target protein‐RNA complexes were captured using either an IgG isotype control antibody or a pan‐AGO antibody. Following complex formation, Proteinase K digestion was performed under specific conditions to release bound RNA. The extracted RNA was purified via phenol:chloroform:isoamyl alcohol extraction to effectively remove impurities. Finally, the RNA was precipitated, concentrated, and treated with DNase I to eliminate genomic DNA contamination, yielding high‐purity RNA suitable for downstream analyses.

### 4.17 Statistical Analysis

All data are expressed as mean ± standard deviation (Mean ± SD). Statistical analyses were conducted using GraphPad Prism software (version 8.0.2). Inter‐group comparisons between two groups utilized an unpaired, two‐tailed Student's t‐test. Comparisons among multiple groups employed either one‐way or two‐way analysis of variance (ANOVA), with Tukey's method applied for post hoc multiple comparisons. Statistical significance was defined as a P‐value below 0.05. All graphical data represent results derived from a minimum of three independent biological replicate experiments. Statistical significance symbols are defined as follows: ns denotes non‐significance (P ≥ 0.05), * P < 0.05, ** P < 0.01, *** P < 0.001.

## Author Contributions

**Wu Xiong** was primarily responsible for manuscript drafting, experimental design, and immunofluorescence assays. **Jin Fan** and **Yongjie Zhang** made substantial contributions to the overall study design and project coordination. **Chunming Tang** carried out the fabrication and characterization of hydrogels, in addition to performing all in vivo and ex vivo imaging experiments. **Minhao Liu** conducted Western blot analyses and performed statistical processing. **Mingming Zheng** was responsible for flow cytometry experiments and corresponding statistical evaluations. **Jizhou Jia** and **Ziyan Zhu** performed bioinformatics analyses and animal behavioral tests. **Cong Li** conducted the extraction and identification of extracellular vesicles and carried out TEM. **Juan Wang** implemented PCR and ELISA experiments. **Guang Kong** and **Jie Liu** were engaged in data validation. **Wenbo Li** and **Qingyuan Wang** managed the animal ethics compliance and approval process. **Qian Zhu** and **Peiran Chan** performed LFB staining procedures. **Tao Qin** and **Jiayang Wu** oversaw the postoperative care and management of the experimental mice.

## Funding

This research received funding from the National Natural Science Foundation (82272499, 82571580), Jiangsu Provincial Basic Research Program Climbing Project (BK20250007).

## Ethics Statement

We affirm that all research in this manuscript involving experimental animals was carried out with ethical approval from relevant authorities, and these approvals are acknowledged in the manuscript.

## Consent

All authors consent to the publication.

## Conflicts of Interest

The authors declare no conflicts of interest.

## Supporting information




**Supporting File 1**: advs77117‐sup‐0001‐Figures.docx.

**Supporting File 2**: advs77117‐sup‐0002‐TableS1.xlsx.

## Data Availability

The data that support the findings of this study are available from the corresponding author upon reasonable request.

## References

[1] H. Shen , B. Xu , C. Yang , et al., “A DAMP‐Scavenging, IL‐10‐Releasing Hydrogel Promotes Neural Regeneration and Motor Function Recovery After Spinal Cord Injury,” Biomaterials 280 (2022): 121279. 34847433 10.1016/j.biomaterials.2021.121279

[2] M. A. Anderson , J. W. Squair , M. Gautier , et al., “Natural and Targeted Circuit Reorganization after Spinal Cord Injury,” Nature Neuroscience 25, no. 12 (2022): 1584–1596. 36396975 10.1038/s41593-022-01196-1

[3] C. Fu , X. Jin , K. Ji , et al., “Macrophage‐Targeted Mms6 mRNA‐Lipid Nanoparticles Promote Locomotor Functional Recovery After Traumatic Spinal Cord Injury in Mice,” Science Advances 11, no. 13 (2025): ads2295. 10.1126/sciadv.ads2295 PMC11939073 40138430

[4] F. H. Brennan , Y. Li , C. Wang , et al., “Microglia Coordinate Cellular Interactions During Spinal Cord Repair in Mice,” Nature Communications 13, no. 1 (2022): 4096. 10.1038/s41467-022-31797-0 PMC9283484 35835751

[5] C. Li , Z. Wu , L. Zhou , et al., “Temporal and Spatial Cellular and Molecular Pathological Alterations With Single‐Cell Resolution in the Adult Spinal Cord After Injury,” Signal Transduction and Targeted Therapy 7, no. 1 (2022): 65. 35232960 10.1038/s41392-022-00885-4 PMC8888618

[6] Y.‐Q. Wu , J. Xiong , Z.‐L. He , et al., “Metformin Promotes Microglial Cells to Facilitate Myelin Debris Clearance and Accelerate Nerve Repairment After Spinal Cord Injury,” Acta Pharmacologica Sinica 43, no. 6 (2022): 1360–1371. 34480113 10.1038/s41401-021-00759-5 PMC9160053

[7] M. Luo , J. Liu , Y. Su , et al., “Regulating PPARG Reduces Lipid Accumulation in Microglia and Promotes Functional Recovery After Spinal Cord Injury,” Advanced Science 12, no. 43 (2025): 06313. 10.1002/advs.202506313 PMC12631859 40940667

[8] Y. Liu , F. Yao , Z. Li , et al., “Dynamic Phosphorylation of Fascin‐1 Orchestrates Microglial Phagocytosis and Neurological Recovery After Spinal Cord Injury,” Journal of Neuroinflammation 22, no. 1 (2025): 121. 40281563 10.1186/s12974-025-03445-z PMC12032802

[9] W. Xiao , W. Jiang , Z. Chen , et al., “Advance in Peptide‐Based Drug Development: Delivery Platforms, Therapeutics and Vaccines,” Signal Transduction and Targeted Therapy 10, no. 1 (2025): 74. 40038239 10.1038/s41392-024-02107-5 PMC11880366

[10] Y. Ju , Y. Hu , P. Yang , X. Xie , and B. Fang , “Extracellular Vesicle‐Loaded Hydrogels for Tissue Repair and Regeneration,” Materials Today Bio 18 (2023): 100522. 10.1016/j.mtbio.2022.100522 PMC9803958 36593913

[11] M. M. Lino , S. Simões , F. Tomatis , et al., “Engineered Extracellular Vesicles as Brain Therapeutics,” Journal of Controlled Release 338 (2021): 472–485. 34428481 10.1016/j.jconrel.2021.08.037

[12] N. Ji , F. Wang , M. Wang , W. Zhang , H. Liu , and J. Su , “Engineered Bacterial Extracellular Vesicles for Central Nervous System Diseases,” Journal of Controlled Release 364 (2023): 46–60. 37866404 10.1016/j.jconrel.2023.10.027

[13] T. Qin , Y. Qin , H. Wen , et al., “Hypoxic Neural Stem Cells Enhance Spinal Cord Repair Through HIF‐1a/RAB17‐Driven Extracellular Vesicle Release,” Journal of Extracellular Vesicles 14, no. 7 (2025): 70126. 10.1002/jev2.70126 PMC12259395 40660091

[14] J. Lin , Q. Wang , S. Zhou , S. Xu , and K. Yao , “Tetramethylpyrazine: A Review on its Mechanisms and Functions,” Biomed Pharmacother 150 (2022): 113005. 35483189 10.1016/j.biopha.2022.113005

[15] Z. Meng , H. Chen , and S. Meng , “The Roles of Tetramethylpyrazine During Neurodegenerative Disease,” Neurotoxicity Research 39, no. 5 (2021): 1665–1677. 34351568 10.1007/s12640-021-00398-y

[16] M. Qi , X. Su , Z. Li , et al., “Bibliometric Analysis of Research Progress on Tetramethylpyrazine and its Effects on Ischemia‐Reperfusion Injury,” Pharmacology & Therapeutics 259 (2024): 108656. 38735486 10.1016/j.pharmthera.2024.108656

[17] J. Li , J. Wei , Y. Wan , et al., “TAT‐Modified Tetramethylpyrazine‐Loaded Nanoparticles for Targeted Treatment of Spinal Cord Injury,” Journal of Controlled Release 335 (2021): 103–116. 34015402 10.1016/j.jconrel.2021.05.016

[18] L. Jiang , C.‐L. Pan , C.‐Y. Wang , et al., “Selective Suppression of the JNK‐MMP2/9 Signal Pathway by Tetramethylpyrazine Attenuates Neuropathic Pain in Rats,” Journal of Neuroinflammation 14, no. 1 (2017): 174. 28859670 10.1186/s12974-017-0947-x PMC5580313

[19] L. Chen , W. Hong , W. Ren , T. Xu , Z. Qian , and Z. He , “Recent Progress in Targeted Delivery Vectors Based on Biomimetic Nanoparticles,” Signal Transduction and Targeted Therapy 6, no. 1 (2021): 225. 34099630 10.1038/s41392-021-00631-2 PMC8182741

[20] Z. Li , S. Jiang , J. Wang , et al., “Peptide‐Drug Conjugates Repolarize Glioblastoma‐Associated Macrophages to Resensitize Chemo‐Immunotherapy of Glioblastoma,” Science Advances 11, no. 3 (2025): adr8841. 10.1126/sciadv.adr8841 PMC11740939 39823328

[21] G. Kong , J. Liu , J. Wang , et al., “Engineered Extracellular Vesicles Modified by Angiopep‐2 Peptide Promote Targeted Repair of Spinal Cord Injury and Brain Inflammation,” ACS Nano 19, no. 4 (2025): 4582–4600. 39853366 10.1021/acsnano.4c14675 PMC11803916

[22] B. H. Shan and F. G. Wu , “Hydrogel‐Based Growth Factor Delivery Platforms: Strategies and Recent Advances,” Advanced Materials 36, no. 5 (2024): 2210707. 10.1002/adma.202210707 37009859

[23] Y. Wang , H. Q. Lv , X. Chao , et al., “Multimodal Therapy Strategies Based on Hydrogels for the Repair of Spinal Cord Injury,” Military Medical Research 9 (2022): 16. 35410314 10.1186/s40779-022-00376-1 PMC9003987

[24] C. M. Walsh , J. K. Wychowaniec , D. F. Brougham , and D. Dooley , “Functional Hydrogels as Therapeutic Tools for Spinal Cord Injury: New Perspectives on Immunopharmacological Interventions,” Pharmacology & Therapeutics 234 (2022): 108043. 34813862 10.1016/j.pharmthera.2021.108043

[25] M. Pu , H. Cao , H. Zhang , et al., “ROS‐Responsive Hydrogels: From Design and Additive Manufacturing to Biomedical Applications,” Materials Horizons 11 (2024): 3721–3746. 38894682 10.1039/d4mh00289j

[26] W. Xiong , M. Liu , J. Wang , et al., “Multidimensional Engineering of Extracellular Vesicles for Targeted Delivery and Microglial Reprograming in Spinal Cord Injury Repair,” ACS Nano 19, no. 35 (2025): 31551–31571. 40845835 10.1021/acsnano.5c08573 PMC12424966

[27] T.‐C. Tseng , L. Tao , F.‐Y. Hsieh , Y. Wei , I.‐M. Chiu , and S.‐H. Hsu , “An Injectable, Self‐Healing Hydrogel to Repair the Central Nervous System,” Advanced Materials 27, no. 23 (2015): 3518–3524. 25953204 10.1002/adma.201500762

[28] L. Fan , C. Liu , X. Chen , et al., “Directing Induced Pluripotent Stem Cell Derived Neural Stem Cell Fate With a Three‐Dimensional Biomimetic Hydrogel for Spinal Cord Injury Repair,” ACS Applied Materials & Interfaces 10, no. 21 (2018): 17742–17755. 29733569 10.1021/acsami.8b05293

[29] T. J. Kopper and J. C. Gensel , “Myelin as an Inflammatory Mediator: Myelin Interactions With Complement, Macrophages, and Microglia in Spinal Cord Injury,” Journal of Neuroscience Research 96, no. 6 (2018): 969–977. 28696010 10.1002/jnr.24114 PMC5764830

[30] S. A. Berghoff , L. Spieth , T. Sun , et al., “Microglia Facilitate Repair of Demyelinated Lesions via Post‐Squalene Sterol Synthesis,” Nature Neuroscience 24, no. 1 (2021): 47–60. 33349711 10.1038/s41593-020-00757-6 PMC7116742

[31] B. Lin , Y. Zhou , Z. Huang , et al., “GPR34 Senses Demyelination to Promote Neuroinflammation and Pathologies,” Cellular & Molecular Immunology 21, no. 10 (2024): 1131–1144. 39030423 10.1038/s41423-024-01204-3 PMC11442997

[32] L.‐Q. Zhou , M.‐H. Dong , Z.‐W. Hu , et al., “Staged Suppression of Microglial Autophagy Facilitates Regeneration in CNS Demyelination by Enhancing the Production of Linoleic Acid,” Proceedings of the National Academy of Sciences U S A 120, no. 1 (2023): 2209990120. 10.1073/pnas.2209990120 PMC9910603 36577069

[33] J. Van Broeckhoven , D. Sommer , D. Dooley , S. Hendrix , and A. Franssen , “Macrophage Phagocytosis after Spinal Cord Injury: When Friends Become Foes,” Brain 144 (2021): 2933–2945. 34244729 10.1093/brain/awab250

[34] R. Berglund , A. O. Guerreiro‐Cacais , M. Z. Adzemovic , et al., “Microglial Autophagy–Associated Phagocytosis is Essential for Recovery From Neuroinflammation,” Science Immunology 5, no. 52 (2020): eabb5077. 33067381 10.1126/sciimmunol.abb5077

[35] X. Yu , Y. C. Long , and H. M. Shen , “Differential Regulatory Functions of Three Classes of Phosphatidylinositol and Phosphoinositide 3‐kinases in Autophagy,” Autophagy 11, no. 10 (2015): 1711–1728. 26018563 10.1080/15548627.2015.1043076 PMC4824607

[36] Z. Li , H. Yu , Z. Wang , et al., “Recent Advances in Nanotechnology for Repairing Spinal Cord Injuries,” Biomaterials 323 (2025): 123422. 40403446 10.1016/j.biomaterials.2025.123422

[37] Y. Ma , X. Yu , J. Pan , et al., “Exosomes: A Promising Microenvironment Modulator for Spinal Cord Injury Treatment,” International Journal of Biological Sciences 21, no. 8 (2025): 3791–3824. 40520019 10.7150/ijbs.115242 PMC12160932

[38] R. Kalluri and V. S. LeBleu , “The Biology, Function, and Biomedical Applications of Exosomes,” Science 367 (2020): aau6977. 10.1126/science.aau6977 PMC7717626 32029601

[39] R. Wang , X. Wang , Y. Zhang , et al., “Emerging Prospects of Extracellular Vesicles for Brain Disease Theranostics,” Journal of Controlled Release 341 (2022): 844–868. 34953980 10.1016/j.jconrel.2021.12.024

[40] Z. Lv , J. Xu , X. Cai , et al., “Exosome‐Based Therapeutics for Musculoskeletal Disorders: Advances in Engineering, Targeting, and Biomaterial Integration,” ACS Nano 19, no. 38 (2025): 33681–33716. 40963099 10.1021/acsnano.5c05416 PMC12490021

[41] M. Ala , “The Beneficial Effects of Mesenchymal Stem Cells and Their Exosomes on Myocardial Infarction and Critical Considerations for Enhancing Their Efficacy,” Ageing Research Reviews 89 (2023): 101980. 37302757 10.1016/j.arr.2023.101980

[42] Y. Hu , R. Tao , L. Chen , et al., “Exosomes Derived from Pioglitazone‐Pretreated MSCs Accelerate Diabetic Wound Healing Through Enhancing Angiogenesis,” Journal of Nanobiotechnology 19, no. 1 (2021): 150. 34020670 10.1186/s12951-021-00894-5 PMC8139165

[43] S. Li , K. Yang , W. Cao , et al., “Tanshinone IIA Enhances the Therapeutic Efficacy of Mesenchymal Stem Cells Derived Exosomes in Myocardial Ischemia/Reperfusion Injury via Up‐Regulating miR‐223‐5p,” Journal of Controlled Release 358 (2023): 13–26. 37086952 10.1016/j.jconrel.2023.04.014

[44] Y. Liang , L. Duan , J. Lu , and J. Xia , “Engineering Exosomes for Targeted Drug Delivery,” Theranostics 11, no. 7 (2021): 3183–3195. 33537081 10.7150/thno.52570 PMC7847680

[45] S. Zhao , Y. Di , H. Fan , et al., “Targeted Delivery of Extracellular Vesicles: The Mechanisms, Techniques and Therapeutic Applications,” Molecular Biomedicine 5, no. 1 (2024): 60. 39567444 10.1186/s43556-024-00230-x PMC11579273

[46] S. Salunkhe , M. B. Dheeraj , D. Chitkara , and A. Mittal , “Surface Functionalization of Exosomes for Target‐Specific Delivery and In Vivo Imaging & Tracking: Strategies and Significance,” Journal of Controlled Release 326 (2020): 599–614. 32730952 10.1016/j.jconrel.2020.07.042

[47] Y. Rong , et al., “Engineered Extracellular Vesicles for Delivery of siRNA Promoting Targeted Repair of Traumatic Spinal Cord Injury,” Bioactive Materials 23 (2023): 328–342. 36474657 10.1016/j.bioactmat.2022.11.011 PMC9706413

[48] Y. Xie , Y. Sun , Y. Liu , et al., “Targeted Delivery of RGD‐CD146^+^ CD271^+^ Human Umbilical Cord Mesenchymal Stem Cell‐Derived Exosomes Promotes Blood–Spinal Cord Barrier Repair After Spinal Cord Injury,” ACS Nano 17, no. 18 (2023): 18008–18024. 37695238 10.1021/acsnano.3c04423

[49] E. J. Roh , D.‐S. Kim , J. H. Kim , et al., “Multimodal Therapy Strategy Based on a Bioactive Hydrogel for Repair of Spinal Cord Injury,” Biomaterials 299 (2023): 122160. 37209541 10.1016/j.biomaterials.2023.122160

[50] L. Fan , C. Liu , X. Chen , et al., “Exosomes‐Loaded Electroconductive Hydrogel Synergistically Promotes Tissue Repair After Spinal Cord Injury via Immunoregulation and Enhancement of Myelinated Axon Growth,” Advanced Science 9, no. 13 (2022): 2105586. 35253394 10.1002/advs.202105586 PMC9069372

[51] C. Wang , M. Wang , K. Xia , et al., “A Bioactive Injectable Self‐Healing Anti‐Inflammatory Hydrogel With Ultralong Extracellular Vesicles Release Synergistically Enhances Motor Functional Recovery of Spinal Cord Injury,” Bioactive Materials 6 (2021): 2523–2534. 33615043 10.1016/j.bioactmat.2021.01.029 PMC7873581

[52] J. Cao , X. Zhang , J. Guo , et al., “An Engineering‐Reinforced Extracellular Vesicle–Integrated Hydrogel With an ROS‐Responsive Release Pattern Mitigates Spinal Cord Injury,” Science Advances 11, no. 14 (2025): ads3398. 10.1126/sciadv.ads3398 PMC11963969 40173229

[53] Y. Zhou , T. Xu , Y. Zhou , et al., “A Myelin Debris Cleaner for Spinal Cord Injury Recovery: Polycaprolactone /Cell Membrane Assembled Scaffolds,” Advanced Science 12, no. 36 (2025): 03269. 10.1002/advs.202503269 PMC12463047 40568983

[54] C. Y. Chung , et al., “Inhibition of the PI3K‐AKT‐MTORC1 Axis Reduces the Burden of the m.3243A>G mtDNA Mutation by Promoting Mitophagy and Improving Mitochondrial Function,” Autophagy 21 (2025): 881–896. 39667405 10.1080/15548627.2024.2437908 PMC11925111

[55] R. Song , S. He , Y. Cao , et al., “Cadmium Accelerates Autophagy of Osteocytes by Inhibiting the PI3K/AKT/mTOR Signaling Pathway,” Environmental Toxicology 38, no. 8 (2023): 1980–1988. 37148155 10.1002/tox.23823

[56] M. Kundu , S. Das , C. K. Das , et al., “Magnolol Induces Cytotoxic Autophagy in Glioma by Inhibiting PI3K/AKT/mTOR Signaling,” Experimental Cell Research 424, no. 1 (2023): 113488. 36736226 10.1016/j.yexcr.2023.113488

[57] Y. Gao , Y. Zhang , and Y. Fan , “Eupafolin Ameliorates Lipopolysaccharide‐Induced Cardiomyocyte Autophagy via PI3K/AKT/mTOR Signaling Pathway,” Iranian Journal of Basic Medical Sciences 22 (2019): 1340–1346. 32128100 10.22038/ijbms.2019.37748.8977 PMC7038429

[58] E. A. Turovsky , V. V. Golovicheva , E. G. Varlamova , et al., “Mesenchymal Stromal Cell‐Derived Extracellular Vesicles Afford Neuroprotection by Modulating PI3K/AKT Pathway and Calcium Oscillations,” International Journal of Biological Sciences 18, no. 14 (2022): 5345–5368. 36147480 10.7150/ijbs.73747 PMC9461662

[59] J. Zuo , Q. Guo , X. Fu , Z. Li , F. Zhang , and J. Zheng , “Stem Cells of Human Exfoliated Deciduous Teeth ‐Delivered miR‐200c‐3p Contained in Small Extracellular Vesicles Regulates M2 Macrophage Polarization via PTEN/PI3K/Akt Pathway In Vitro,” Biochemical and Biophysical Research Communications 777 (2025): 152259. 40618438 10.1016/j.bbrc.2025.152259

[60] Y. Guo , H. Wu , J. Xiong , S. Gou , J. Cui , and T. Peng , “miR‐222‐3p‐Containing Macrophage‐Derived Extracellular Vesicles Confer Gemcitabine Resistance via TSC1‐Mediated mTOR/AKT/PI3K Pathway in Pancreatic Cancer,” Cell Biology and Toxicology 39, no. 4 (2023): 1203–1214. 35974258 10.1007/s10565-022-09736-y

