# Supplementary material for: A Multidimensional Engineering Strategy Reprograms Microglia via Targeted and Sustained‐Release Extracellular Vesicles for Spinal Cord Injury Repair
Source: Adv Sci (Weinh). 2026 Aug 13;13(61):e77117. doi: 10.1002/advs.77117 (PMC13472118; doi:10.1002/advs.77117)
Supplement: Supplementary file 1 [file ADVS-13-e77117-s001.docx]

## A Multidimensional Engineering Strategy Reprograms Microglia via Targeted and Sustained-Release Extracellular Vesicles for Spinal Cord Injury Repair

Wu Xiong^1*^, Minhao Liu^1*^, Mingming Zheng^2*^, Jizhou Jia^2^, Ziyan Zhu^2^, Guang Kong^3^, Jie Liu^4^, Juan Wang^2^, Wenbo Li^1^, Qingyuan Wang^1^, Qian Zhu^2^, Peiran Chan^2^, Tao Qin^1^, Jiayang Wu^1^, Yongjie Zhang^2#^, Chunming Tang^5#^, Cong Li^1#^, Jin Fan^1#^

^1^Department of Orthopedics, The First Affiliated Hospital of Nanjing Medical University, 300 Guangzhou Road, Nanjing, Jiangsu, China.

^2^Department of human anatomy, School of Basic Medicine, Nanjing Medical University, Nanjing, Jiangsu, China.

^3^Department of Orthopaedics, Fourth Military Medical University, Xi'an, Shaanxi, China.

^4^Department of Orthopedics, The Affiliated Taizhou People's Hospital of Nanjing Medical University, Taizhou School of Clinical Medicine, Nanjing Medical University, 366 Taihu Road, Taizhou, Jiangsu, China.

^5^Department of Pharmaceutics, School of Pharmacy, Nanjing Medical University, 300 Guangzhou Road, Nanjing, Jiangsu, China.

^*^Wu Xiong , Minhao Liu , and Mingming Zheng contributed equally to this work.

^#^Corresponding Author:

Jin Fan Email: [fanjin@njmu.edu.cn](mailto:fanjin@njmu.edu.cn)

Cong Li Email:licong@njmu.edu.cn

Chunming Tang Email:[cmtang@njmu.edu.cn](mailto:cmtang@njmu.edu.cn)

Yongjie Zhang Email:zhangyongjie@njmu.edu.cn

Figure S1. (A-B) Ex vivo organ imaging results and quantitative fluorescence intensity analysis of mice treated with TEVs or Ang-TEVs (n = 3).

Figure S2. (A) Rheological property of Ang-TEVs@Gel. Storage modulus (G’) and loss modulus (G’’) of Ang-TEVs@Gel. (B) Swelling behavior of Ang-TEVs@Gel. (C) Degradation behavior of Ang-TEVs@Gel. (D-G) Batch-to-batch reproducibility of Ang-TEVs@Gel, including the rheological property (D), gelation time (E), swelling ratio (F) and degradation rate (G). Results are reported as mean ± SD (n=3).

Figure S3. (A-B) Representative DHE fluorescence images and quantitative fluorescence analysis (n = 3). Scale bar, 20 μm.

Figure S4. (A-B) Representative transmission electron microscopy (TEM) images and quantification of autophagosome numbers (n=3). Scale bar, 2μm. (C-D) Immunoblot analysis of LC3-II and p62 protein levels and quantification (n=3).

Figure S5. (A-B) Representative immunofluorescence images showing the colocalization expression of iNOS (red) or Arg1 (red) with IBA-1 (green), and quantitative analysis results (n=3). Scale bar, 20 μm.
